# Pan-Arctic marine biodiversity and species co-occurrence patterns under recent climate

**DOI:** 10.1038/s41598-023-30943-y

**Published:** 2023-03-11

**Authors:** Irene D. Alabia, Jorge García Molinos, Takafumi Hirata, Franz J. Mueter, Carmen L. David

**Affiliations:** 1 Arctic Research Center, Hokkaido University, N21 W11, Kita-Ku, Sapporo, Hokkaido 001-0021 Japan; 2 College of Fisheries and Ocean Sciences, University of Alaska Fairbanks, 17101 Point Lena Loop Rd, 315 Lena Point Bldg, Juneau, AK 99801-8344 USA; 3 Wageningen University and Research, Wageningen, 6708 PB The Netherlands

**Keywords:** Climate-change ecology, Biodiversity, Marine biology, Biooceanography, Boreal ecology

## Abstract

The Arctic region is experiencing drastic climatic changes bringing about potential ecological shifts. Here, we explored marine biodiversity and potential species associations across eight Arctic marine areas between 2000 and 2019. We compiled species occurrences for a subset of 69 marine taxa (i.e., 26 apex predators and 43 mesopredators) and environmental factors to predict taxon-specific distributions using a multi-model ensemble approach. Arctic-wide temporal trends of species richness increased in the last 20 years and highlighted potential emerging areas of species accrual due to climate-driven species redistribution. Further, regional species associations were dominated by positive co-occurrences among species pairs with high frequencies in the Pacific and Atlantic Arctic areas. Comparative analyses of species richness, community composition, and co-occurrence between high and low summer sea ice concentrations revealed contrasting impacts of and detected areas vulnerable to sea ice changes. In particular, low (high) summer sea ice generally resulted in species gains (loss) in the inflow and loss (gains) in the outflow shelves, accompanied by substantial changes in community composition and therefore potential species associations. Overall, the recent changes in biodiversity and species co-occurrences in the Arctic were driven by pervasive poleward range shifts, especially for wide-ranging apex predators. Our findings highlight the varying regional impacts of warming and sea ice loss on Arctic marine communities and provide important insights into the vulnerability of Arctic marine areas to climate change.

## Introduction

Over the recent decades, the Arctic region has experienced unprecedented anthropogenic climate changes. The extent and magnitude of climate fluctuations often vary across space and persist at different timescales. For instance, the on-going ocean warming and sea ice loss in the Arctic Ocean substantially alter its physical and biogeochemical properties, leading to notable changes in nutrient fluxes^1^, material transport^2^, and consequent increases in primary production^3,4^. Moreover, these environmental changes in the Arctic have evident impacts on marine ecosystems through biogeographical shifts and reorganization of marine communities and biodiversity^5,6^. Nonetheless, our current knowledge of Arctic marine communities and stressors remains scarce and limits our understanding of trends and their implications on biodiversity aspects^7^. Here, we explore the recent changes in species richness, composition and potential species associations on a pan-Arctic scale. In doing so, we hope to augment information on climate-driven Arctic biodiversity responses and deduce pertinent ecological implications of these changes in the recent past.

In particular, the northward expansion of temperate marine taxa, especially along the continental margins and within the major inflow shelves in the Pacific (Bering-Chukchi seas) and Atlantic (Barents Sea) sectors has been facilitated by recent record-breaking sea ice loss and retreat, ocean warming, and enhanced productivity in the Arctic regions^8,9^. In both of these inflow shelves, poleward shifts of warm-affinity taxa and highly-migratory apex predators have promoted the borealization and consequent restructuring of the Arctic marine ecosystem, driving significant changes in the community structure, functional biogeography and biodiversity facets^6,8,10,11^. The Arctic outflow shelves (Canadian Arctic Archipelago, East Greenland shelf and Fram Strait), serving as conduits in the export of Arctic sea ice and waters to the North Atlantic Ocean, have similarly shown climate-driven changes in the phytoplankton community composition^12,13^ and distribution of several keystone species supporting northern indigenous communities^14^.

Increasing signals of borealization and biodiversity changes, in turn, result in potential changes in species interactions either through the formation of novel or loss of existing species interactions. For instance, the frequency and strength of species interactions in the community vary with biodiversity^15^ and determine the stability of natural ecosystems^16^. Understanding species interactions is therefore crucial for ecological studies as they create the basis for ecosystem properties and processes affecting community responses to disturbance. One of the approaches to infer potential ecological interactions is through analyses of spatial patterns in pairwise species co-occurrences (presence–absence). This approach is long debated^17^ in relation to arguments that systematic analysis of pairwise co-occurrences to determine the degree of associations between species pairs is strongly dependent on the spatial scale of the sampling unit^18^. It is also argued to be dependent on the regularity of signal of ecological interactions that permit their detection and interpretation using adequate statistical methods^19^. At the core of this approach is the ecological truism that species have to co-occur for them to have direct interactions and influence the occurrence of one another. These species interactions are essential for understanding the patterns and drivers of community assembly^20,21^. In general, the non-random patterns of species co-occurrence could result from habitat overlap, dispersal limitations, and biotic interactions, consequently allowing some species to coexist more or less often than expected by chance^20,22^. Many of these species co-occurrences may be vital to maintaining community structure and function and therefore constitute relevant aspects of biodiversity conservation^23^.

The preponderance of evidence highlighting the differences in physical and biological structures among diverse marine ecosystems of the Arctic^5,24^ underpins the need to elucidate the regional climate-driven ecological impacts on marine communities. Comparing Arctic-wide signals could identify potential hotspots of climate and productivity changes and help understand their repercussions on marine biodiversity and potential species associations. Thus, the primary objective of this work is to examine the changes in biodiversity and potential species associations inferred from pairwise co-occurrences in response to climatic fluctuations and food availability in the Arctic during the last two decades. Our analyses were implemented using spatial distributions of 26 apex and 43 mesopredatory sub-Arctic and Arctic taxa derived from species distribution models. Focusing on a modest subset of species from these distinct communities allowed us to evaluate differential biodiversity responses between the high (apex predators) and mid-trophic (mesopredators) components of the Arctic marine food webs. Apex predators in the Arctic are highly vulnerable to climate change^25,26^ and the poleward redistribution of mesopredators could further amplify their vulnerability through changes in species associations. To our knowledge, our work is the first attempt to explore Arctic-wide potential patterns of species associations due to climate- and productivity-driven changes in biodiversity. In particular, the Arctic summer sea ice is shrinking by almost 13% per decade, with precipitous declines observed in recent years (2000–2019)^27^. These changes in the Arctic sea ice environment have serious ecological consequences^28,29^, many of which remain poorly understood. A comparison of Arctic community responses to changing sea ice could provide insights into the vulnerability of individual species and communities. Thus, our specific aim is to examine spatial patterns and temporal trends of species richness, composition, and potential species associations of Arctic marine communities over the last 20 years and between periods of high and low summer sea ice extent from 2000 to 2019.

## Results

### Marine community responses during the recent decades (2000–2019)

#### Spatial patterns and temporal trends of species richness

**Figure 1:**
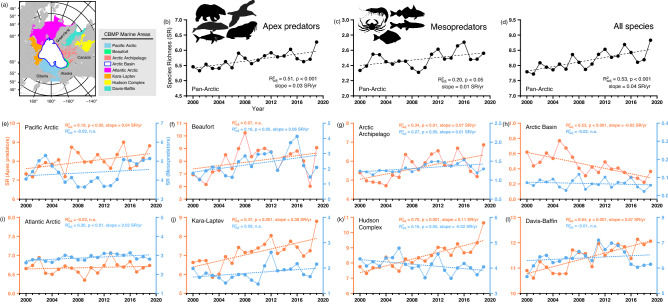
(**a**) Geographic map of the study area and annual time-series of predicted pan-Arctic species richness for (**b**) apex predators, (**c**) mesopredators, and (**d**) all species combined. Second and third row of panels show regional species richness by year with estimated linear trends over time for apex predators (orange) and mesopredators (blue) in the (**e**) Pacific Arctic, (**f**) Beaufort, (**g**) Arctic Archipelago, (**h**) Arctic Basin, (**i**) Atlantic Arctic, (**j**) Kara–Laptev, (**k**) Hudson complex, and (**l**) Davis-Baffin between 2000 and 2019. The map in (**a**) was created using GMT 6.3.0. (https://docs.generic-mapping-tools.org/6.3/gmt.html).

During the last two decades, the pan-Arctic (averaged across all CBMP areas; Fig. 1a) species richness (SR) for apex predators, mesopredators, and all taxa combined increased (Fig. 1b–d). Regionally, increasing trends in overall species richness were seen in the Arctic Archipelago, Kara-Laptev, Hudson Complex, and Davis-Baffin and a decreasing trend for the Arctic Basin (Fig. S1). The regional SR computed for each marine community (Fig. 1e–l), showed that trends in overall richness are accounted for by changes in apex predators. In particular, a significant decrease and increase in the number of apex predators was noted, respectively, in the Arctic Basin (Fig. 1h) and Hudson Complex (1.1 species/decade; Fig. 1k). For mesopredator richness, significant increases were restricted to the Beaufort (Fig. 1f), Arctic Archipelago (Fig. 1g), and Atlantic Arctic (Fig. 1i). However, a decline in mesopredators emerged in the Hudson Complex (Fig. 1g), effectively offsetting the increase in apex predators over this area. Thus, over the 20-year period the Hudson Complex showed the largest increase and the largest decrease among regions in the number of apex and mesopredators, respectively.

**Figure 2:**
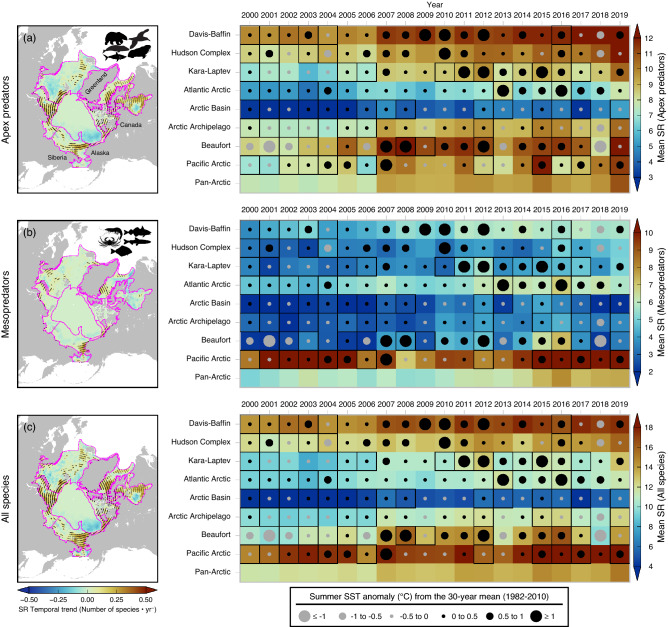
Distribution of temporal trends of species richness (left panels) and Hovmöller plots (right panels) of annual averaged species richness in areas of species accrual (pixels with species gain ≥ 1 species/decade, represented by the black dots in the left panels) for (**a**) apex predators, (**b**) mesopredators, and (**c**) all species from 2000 to 2019. Years with lower than regionally-averaged summer sea ice are indicated in solid squares and scaled magnitudes of summer sea surface anomaly relative to the long-term mean (1982–2011) are represented by shaded circles. The maps were created using GMT 6.3.0 (https://docs.generic-mapping-tools.org/6.3/gmt.html).

Temporal SR trends during the 20-year period further revealed increases in areas along continental shelf breaks, surrounding the Arctic basin (Fig. 2a–c, left panels). In general, apex predators were more diverse in these areas (Fig. 2a, left panel; Fig. S2) relative to mesopredators (Fig. 2b, left panel; Fig. S3). The former substantially accounted for the increase in overall SR (Fig. 2c, left panel; Fig. S4). Potential emerging areas of species accrual from climate-driven species redistribution and defined as areas of increased richness (≥ 1 species/decade) in the last 20 years, featured sharp spatial contrasts (Fig. 2a–c, dotted sites on right panels) and cell-wise frequencies (Table S1) across marine communities and Arctic areas. In particular, apex predators were highest in warm years in Beaufort Sea (10/20 years, Pearson correlation coefficient (*r*) = 0.43) and Davis-Baffin Bay (17/20, *r* = 0.63; Fig. 2a, right panel). However, mesopredators were highest in two major inflow shelves (Pacific and Atlantic Arctic) (Fig. 2b, right panel). In these regions, warm years coincided with accrual of species over time (Pacific Arctic: 13/20, *r* = 0.75; Atlantic Arctic: 20/20, *r* = 0.63). While the Atlantic Arctic has generally warmed during the last 20 years, the number of mesopredators were highest between 2012 and 2019, coinciding with years of moderate and high warming magnitudes (≥ 0.5 °C). Finally, overall were identified in the Pacific Arctic and Davis-Baffin Bay (Fig. 2c, right panel), in response to species gains in warm years.

#### Temporal trends of species co-occurrence

**Figure 3:**
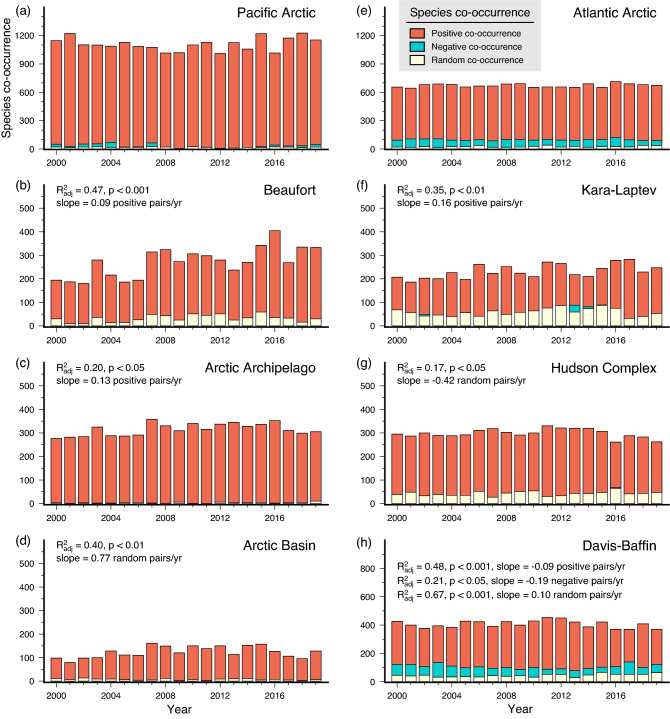
Frequency of positive, negative, and random co-occurrences for all species in the (**a**) Pacific Arctic, (**b**) Beaufort, (**c**) Arctic Archipelago, (**d**) Arctic Basin, (**e**) Atlantic Arctic, (**f**) Kara–Laptev, (**g**) Hudson Complex, and (**h**) Davis-Baffin from 2000 to 2019. Adjusted R^2^ (R^2^_adj_) and slope are shown for areas exhibiting significant temporal trends in species co-occurrences.

Potential associations from co-occurrences between two species were classified into positive, negative and random and showed a predominance of positive co-occurrences across all CBMP areas (Fig. 3a–h). This suggests that most species pairs occur together and are positively associated. The frequency of co-occurring species were highest in the Pacific and Atlantic Arctic, but no significant trends were observed on these inflow shelves during the last 20 years (Fig. 3a,e). In contrast, significant increases in positive species associations over time were observed in the Beaufort (Fig. 3b), Arctic Archipelago (Fig. 3c) and Kara–Laptev (Fig. 3f), with corresponding decline in Davis-Baffin (Fig. 3h). Negative co-occurrences, indicating non-co-occurrence and negative association between two species, were noted for the Atlantic Arctic (Fig. 3e) and Davis-Baffin (Fig. 3h), with a significant decrease in the latter. Random species co-occurrences implying independent distributions between species pairs, increased substantially in the Arctic Basin (Fig. 3d) and Davis-Baffin (Fig. 3h), but declined significantly in the Hudson Complex (Fig. 3g). Correlations between species richness and co-occurrences (positive, negative and random) over the last 20 years were significant in all regions except the Pacific and Atlantic Arctic (Table S2).

### Marine community responses under contrasting sea ice conditions

#### Species richness and composition

**Figure 4:**
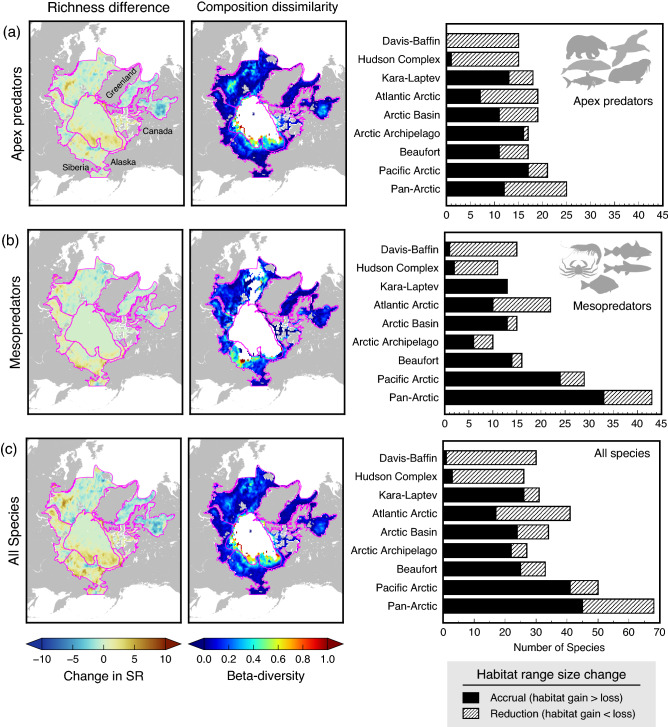
Difference in species richness (low–high sea ice; left panel) and species community composition (middle panels) under contrasting sea ice for (**a**) apex predators, (**b**) mesopredators, and (**c**) all species from 2000 to 2019. Right panels show the number of species exhibiting habitat range size changes between high and low sea ice periods for each Arctic marine area. The maps were created using GMT 6.3.0 (https://docs.generic-mapping-tools.org/6.3/gmt.html).

Differences in species richness of apex, mesopredators, and all species between low and high summer sea ice showed increases near the coasts and continental margins (Fig. 4a–c, left panels). However, decreases in apex predators in the Hudson Complex and Davis-Baffin (Fig. 4a, left panel) drove the overall species declines in these areas during low sea ice (Fig. 4c, left panel). Regions of high mesopredators were nearby shallow areas and southern waters of Pacific Arctic, Atlantic Arctic, and Davis-Baffin (Fig. 4b, left panel). Further, species composition changes between high and low sea ice (Fig. 4a–c, middle panels) and measured using the beta-diversity index, showed different species composition for apex predators in the Arctic Basin and along continental slopes of the Kara-Laptev, Pacific Arctic, and Beaufort seas (Fig. 4a, middle panel). In contrast, highest changes in the species composition of mesopredators were observed west of the Pacific Arctic (Fig. 4b, middle panel). Overall species composition between contrasting sea ice retained high dissimilarity along continental margins and slopes (Fig. 4c, middle panel), and were generally accounted for by the nestedness (i.e., species gain/loss without replacement) rather than the turnover (species replacement) component of beta-diversity (Fig. S5). In general, regional changes in biodiversity measures for apex, mesopredators and all species were driven by gains and losses across different areas of the Arctic due to habitat range size changes during high and low sea ice conditions (Fig. 4a–c, right panels).

#### Species co-occurrence

**Figure 5:**
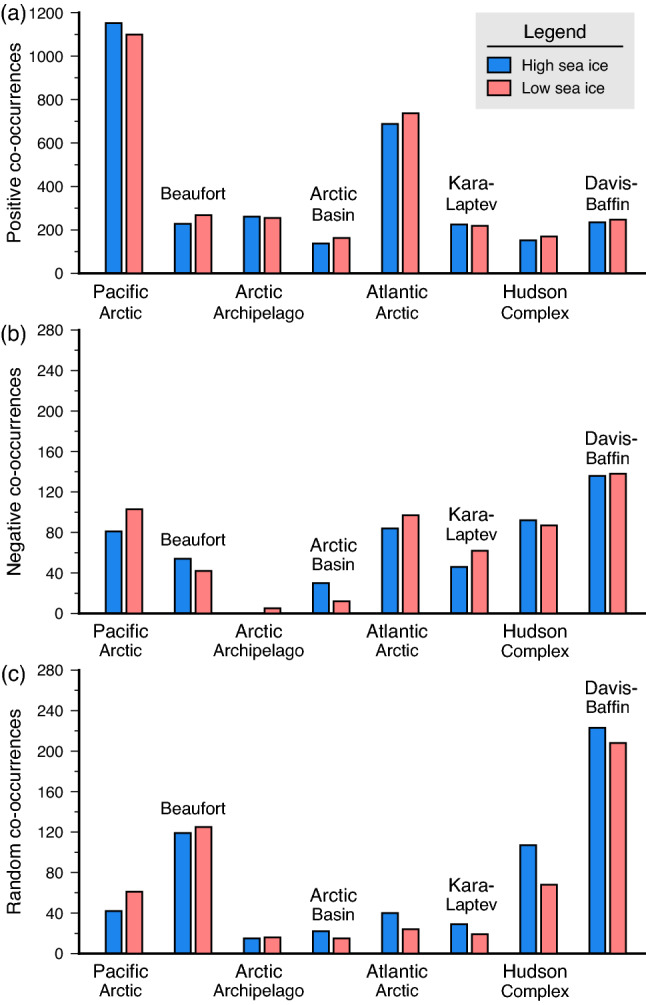
Frequency of (**a**) positive, (**b**) negative, and (**c**) random co-occurrences for all species across the different CBMP areas between periods of high and low summer sea ice conditions from 2000 to 2019.

Frequencies of species co-occurrence (positive, negative, and random) between high and low summer sea ice differed across the Arctic marine areas (Fig. 5a–c). In particular, co-occurrences were largely dominated by co-occurring and positively associated species pairs (positive co-occurrence), with the highest frequency in the Pacific and Atlantic Arctic inflow shelves, albeit with opposite patterns between high and low sea ice (Fig. 5a). For non-co-occurring pairs (negative co-occurrence), however, inflow shelves patterns were consistent and showed more non-co-occurring pairs during low sea ice years (Fig. 5b). Davis-Baffin Bay had the highest frequencies of non-co-occurring (Fig. 5b) and randomly co-occurring species pairs (Fig. 5c) relative to the other CBMP marine areas.

### Climate and productivity hotspots and areas of species accrual

**Figure 6:**
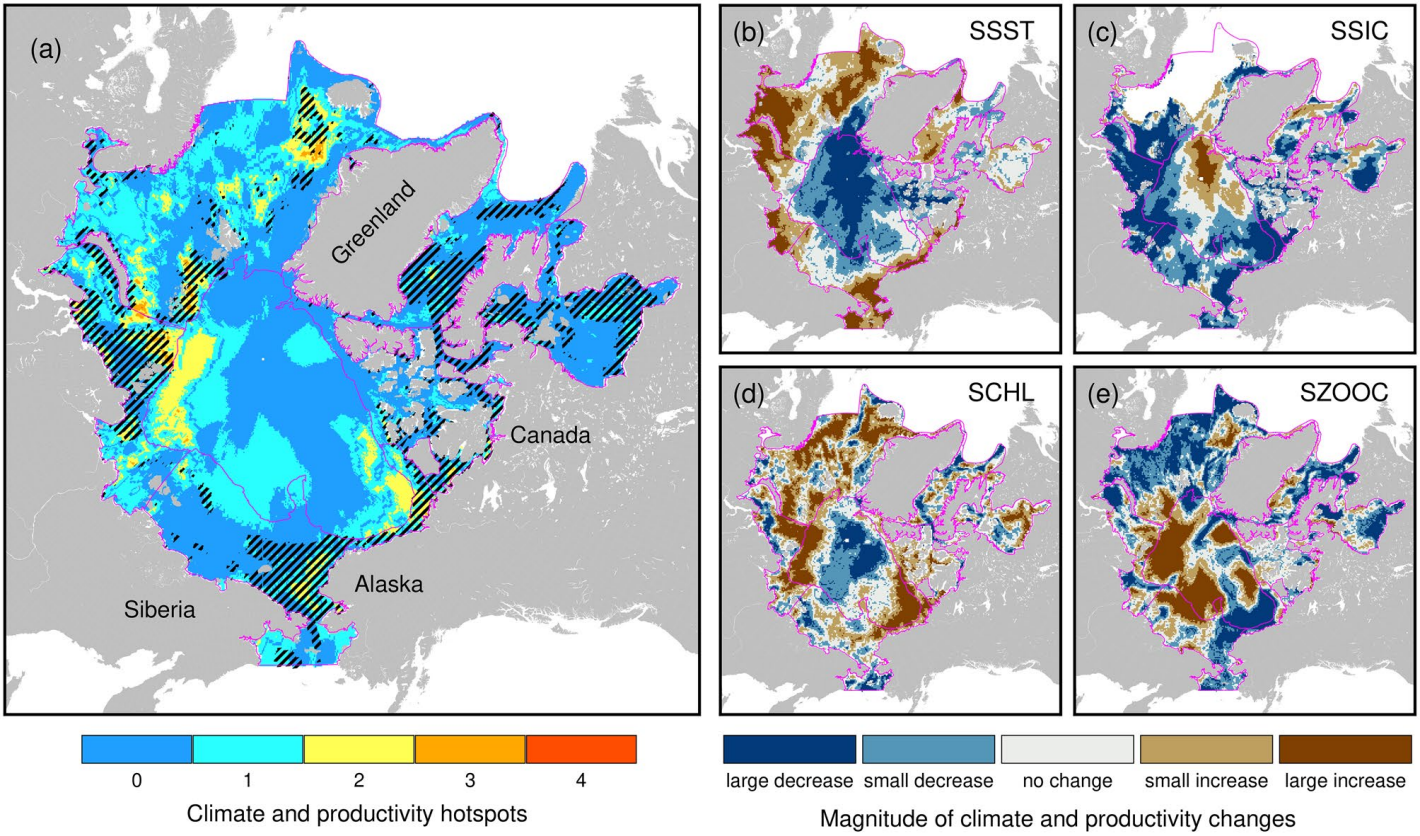
(**a**) Climate and productivity hotspots in Arctic marine areas defined as areas of overlap of two or more large changes in summer (**b**) sea surface temperature (0.21 °C ≤ SSST ≤ 0.76 °C), (**c**) sea ice concentration (− 2% ≥ SSIC ≥ − 76%), (**d**) chlorophyll-a concentration (0.26 mg·m^−3^ ≤ SCHL ≤ 0.73 mg·m^−3^), and (**e**) zooplankton biomass (0.21 g·m^−2^ ≤ SZOOC ≤ 0.75 g·m^−2^). Hatched areas in (**a**) correspond to areas of species accrual (defined as areas with species gains ≥ 1 species/decade) over the entire study period. The maps were created using GMT 6.3.0 (https://docs.generic-mapping-tools.org/6.3/gmt.html).

Climate and productivity hotspots (Fig. 6a), defined as regions where at least 2 of 4 climate and productivity indices suggested warming (large increase in summer sea surface temperature and/or large decrease in sea ice concentration; Fig. 6b,c) or increased productivity (large increases in chlorophyll-*a* and/or zooplankton; Fig. 6d,e), were particularly common in contiguous northern margins of the Kara–Laptev and Atlantic Arctic regions. In the Pacific Arctic, Beaufort and adjacent continental slopes, climate hotspots (high warming and sea ice loss) were similarly identified. High spatial overlap between climate-productivity hotspots and areas of species accrual (increased of ≥ 1 species/decade) were found in Beaufort (31% of pixels), Atlantic Arctic (26%) and Kara–Laptev (19%). The Beaufort Sea had the highest spatial correspondence between sites of high richness and warming (47%, Fig. 6b), sea ice loss (40%, Fig. 6c) and high chlorophyll-*a* concentration (68%, Fig. 6d). In contrast, areas of species accrual in the Atlantic Arctic showed spatial correspondence with sites of warming (47%, Fig. 6b), high chlorophyll-*a* (57%, Fig. 6d) and zooplankton biomass (24%, Fig. 6e). In the Kara–Laptev Sea, species-rich areas corresponded with high changes in all four variables: warming (40%), sea ice loss (26%), chlorophyll-*a* concentration (41%) and zooplankton biomass (28%). While the Hudson Complex and Davis-Baffin Bay showed no apparent climate and productivity hotspots, 42% and 20% of species-rich sites sit atop high summer chlorophyll-*a* waters, respectively.

## Discussion

Our analyses revealed that changes in climate and species richness in the Arctic vary across different large marine areas and highlight potential regions of climate and productivity hotspots, and areas of species accrual. In general, changes are most pronounced across the major inflow and outflow shelves, and along continental slopes and margins. The formation of potential emerging areas of species accrual across the different marine areas were also shown to be variably driven by either climate, productivity, or both. Among all Arctic areas, species accrual in the Kara–Laptev Sea coincided with both climate and productivity hotspots, suggesting high sensitivity to climate and productivity changes over the last two decades. Indeed, this area has experienced one of the highest warming rates and sea ice loss^30^, along with enhanced primary productivity^4^ in recent years. In contrast, other Arctic marine areas including the Davis-Baffin Bay and Hudson Complex, showed no clear overlap between species accrual, climate and productivity hotspots. However, small changes in summer sea temperature and sea ice, along with sustained increases in chlorophyll-*a* concentration, potentially permitted the persistence of areas of species accrual in these waters in the last 20 years. It is important to recognize, however, that our analysis only covers a subset of the boreal and Arctic marine community and may fail to capture the full extent and scale of biodiversity changes among species guilds in different Arctic marine areas. As such, when we refer to changes in species richness or identify hotspots (i.e., areas of species accrual), we implicitly refer only to those corresponding to our study assemblages comprising the pool of species considered for each guild. Our current species pool for mesopredators was primarily comprised of demersal fish and invertebrate species often sampled from bottom trawl surveys and thus, constitutes taxa with the highest number of species records used in model building. The inclusion of a larger species pool of apex and mesopredators in future modeling efforts could provide a more robust representation of marine communities and improved estimates of ongoing biodiversity changes in different areas of the Arctic. For instance, incorporating boreal pelagic fish species in future analyses could capture documented northward range expansions of highly migratory pelagic fish taxa such as Atlantic mackerel^11,31^ and bluefin tuna^32^ into the Arctic waters.

Our analyses of biodiversity between high and low sea ice years revealed prevalent Arctic-wide and regional biodiversity responses under contrasting sea ice conditions. Our findings, along with other studies^8,11,33^, showed sea ice loss facilitated an overall increase in biodiversity in northern Arctic waters, although some regions along the southern limits have shown species declines. For instance, the southern Arctic marine areas such as the Hudson Complex and Davis-Baffin exhibited species decreases in low sea ice, potentially from habitat range size attrition of both apex (e.g., common murre, narwhal, and bowhead whale) and mesopredators (e.g., capelin, Arctic char, and golden redfish). In the Hudson Complex, the quality, concentration, and duration of seasonal sea ice impact the ecology of coastal and ice-associated biota as well as pelagic systems under the ice and at ice edges^34^. In this region, there is little export of particulate organic material out of the euphotic zone and most of it remains in the water column, suggesting a pelagic-dominated food web in summer^35^. This area, along with Davis-Baffin Bay, provides habitat to many ice-associated and seasonally-migrant apex predators such as marine mammals^36,37^ and seabirds^38^. The significant loss of summer sea ice habitats in Arctic waters negatively impact the distributions of top predators through increased energetic costs of long-distance migration^25,39^. Likewise, in the Davis-Baffin Bay, distributions of apex predators were influenced by changes in lifespan, shape, and stability of polynyas, which are important winter sea ice features providing rich foraging environments for many Artic biota^14,40^.

In contrast, in more northern regions (i.e., inflow shelves and the interior shelf and slope regions surrounding the Arctic basin) species increased under low sea ice conditions due to habitat expansions of mesopredators within the shallow shelves and along the margins of the Chukchi Sea and narrow continental Beaufort shelf. This was accompanied by newly available habitat of wide-ranging and highly-mobile apex predators farther away from the coasts, past the continental shelf breaks. These findings are consistent with reported observations of marine apex predators in these waters^41,42^. Moreover, along the inflow shelves of Pacific and Atlantic Arctic, increases in species richness, especially for mesopredators (fish and invertebrates) were in agreement with earlier studies detailing northward expansion of species distributional ranges due to sea ice loss and warming^6,43,44^. However, poleward movements of mesopredators were more limited than those observed for apex predators, suggesting lags in distributional responses between these communities. Potential increases in the primary productivity in these regions in response to reduced stratification, warming, and sea ice loss^3,45,46^, are likely to support elevated secondary production and facilitate the northward habitat range expansions especially for apex predators. Species increases along continental slopes surrounding the Arctic basin are potentially driven by the enhanced gradients of primary productivity and low trophic-level biomass in these areas^47^. In the marginal ice zones, aggregations of zooplankton along the ice edge habitat can further provide prey for highly mobile predators^48^, potentially explaining the richness patterns from model predicted species-specific distributions.

We acknowledge that species co-occurrence patterns identified from our analyses are subject to potential approach-inherent caveats (e.g., scale dependence^18^ and signal regularities of species associations^19^) that may impede the detection of true ecological interactions^17^. Nonetheless, our results provided insights into recent changes in potential species associations that can be attributed to habitat overlap between taxa from different marine communities during unprecedented periods of thermal and sea ice changes. For instance, significant correlations between SR and positive co-occurrences between taxon pairs in the Beaufort, Arctic Archipelago, and Kara–Laptev seas suggest increased species associations due to habitat range expansions and increased overlaps. Increases in positively associated species over time potentially heighten competitions for space and resources, especially among related taxa sharing similar functional morphology or feeding strategies^49^. For instance, enhanced positive co-occurrences among fish taxa due to niche overlap increased prey-predator interactions and interspecific and asymmetric competition^50^. These also generate novel ecological interactions that could have implications for community structure and functioning^51,52^. It is also worth noting the significant drop in species co-occurrences (positive and negative) in the Davis-Baffin Bay over time, implying the potential loss of existing species association and trophic mismatches^53^. Similarly, the concomitant increase in random co-occurrences in this area suggests that most species associations are becoming less environmentally-constrained, potentially due to increases in the environmental heterogeneity and influence of stochastic processes^54,55^. This creates venues for further study on potential community assembly processes driving increases in random species co-occurrences and will allow us to better understand their ecological implications.

Finally, our findings provided Arctic-wide perspectives on marine biodiversity patterns to regional impacts of climate and productivity changes, tapping into large arrays of most recent open-source species and environmental databases. This approach enabled us to incorporate augmented and updated species information into our models to better resolve species distributions in difficult-to-survey waters of the Arctic Ocean. Recognizing the high cost and logistic challenges of conducting sustained surveys in these waters, available species observations in the Arctic remain limited in remote waters. The inclusion of these data when available can improve model predictions and facilitate validation of model-derived species distributions. Nevertheless, our current model-based analyses have reinforced documented biogeographical poleward species shifts in well-studied Arctic marine areas (e.g. Northeastern Bering- Chukchi^8,33^ and Barents^10,11^ seas), potentially altering species associations and creating hotspots (areas of species accrual) in recent decades. The variable geographical extents of habitat range size accrual and loss across different communities also reflect their relative potential to respond to climate and productivity changes and afford insights into species/guild-specific vulnerability. This information is becoming all the more relevant in setting up targets for conservation, management, and sustainable use of Arctic resources, as rates of climate change and its ecosystem impacts are increasingly amplified and anticipated to progress further into the twenty-first century^6,56^.

## Methods

### Study area

Our analyses focused on the eight Arctic marine areas covered by the Circumpolar Biodiversity Monitoring Program (CBMP; https://www.caff.is/monitoring). In the last few decades, these marine areas (Fig. 1a) experienced large climatic changes characterized by pronounced increases in temperature, thinning and loss of sea ice, with important consequences for the structure and function of biological communities and pathways of biogeochemical processes^57,58^. Each of these Arctic marine areas are governed by complex atmospheric and oceanographic processes^59^, regulating aspects of biological productivity and ecosystem structures. In particular, the highly dynamic current systems across the different basins regulate the transport and availability of materials to marine ecosystems^43^.

### Environmental data

We compiled available environmental data from satellite and biogeochemical model outputs from 2000 to 2019 to focus our analyses on the changes over the most recent decades (Table S3). These datasets also included various proxies for climate, bathymetry, and food availability. For sea surface temperature and sea ice concentration, daily AVHRR-OI version 2 data from the NOAA high-resolution blended analysis^60^ were downloaded via file transfer protocol (ftp; ftp://eclipse.ncdc.noaa.gov/pub/OI-daily-v2; date accessed: 06 July 2021) and annual seasonal averages were computed. From the daily sea ice concentration data, we estimated the distance to the summer sea ice edge computed as the shortest distance to the 15% sea ice contour threshold using the ‘raadtools’ R package (https://rdrr.io/github/AustralianAntarcticDivision/raadtools/). Bathymetric variables were obtained from NCDC ETOPO1 Global Relief Model^61^ for depth and NASA Ocean Color (https://oceancolor.gsfc.nasa.gov/docs/distfromcoast/; date accessed: 18 August 2021) for distance to nearest coastline data.

We also compiled outputs from the biogeochemical models for zooplankton, epipelagic micronekton, and chlorophyll-a concentrations as well as salinity to elucidate the contributions of these parameters to species-specific distributions in the study area. The daily zooplankton and epinekton concentration data were sourced from the Global ocean low and mid trophic levels biomass content hindcast (https://doi.org/10.48670/moi-00020)^62^ available and downloaded from the E.U. Copernicus Marine Environment Monitoring Service (CMEMS; date accessed: 19 August 2021). These model outputs have been earlier used for understanding foraging behavior and environmental drivers of large-scale movement of apex predators^63,64^. The monthly chlorophyll-a data were sourced from the Global ocean biogeochemistry hindcast (https://doi.org/10.48670/moi-00019) and downloaded from the CMEMS website (date accessed: 19 August 2021). Monthly salinity data were sourced from the Global Ocean Ensemble Physics Reanalysis (https://doi.org/10.48670/moi-00024; date accessed: 19 August 2021). Spatial distributions of these environmental variables over sustained and gap-free spatial and temporal periods are often unavailable from satellite and observational sources.

### Species presence/pseudo-absence data for species distribution modeling

Annual occurrence data for each species within the study area (− 180 to 180; 50–90 N), downloaded from the different databases, were then merged into annual datasets and were checked for data quality. Unique entries based on the geographic coordinates were retained and occurrences at sea were used for subsequent analyses. To minimize spatial sampling biases in the annually-compiled species-specific occurrences (e.g., Fig. S6), spatial data thinning was conducted using the ‘*thin.algorithm*’ function of spThin (https://rdrr.io/cran/spThin/man/thin.html)^65^ R package. We obtained presence records using different spatial thresholds (25-km, 50-km, and 100-km; Figs. S7–Figs. S9) and selected the 100-km grid size, as it better accounted for spatial autocorrelation and retained the smallest number of samples in each cell relative to other thresholds (Fig. S9).

The species data were downloaded from online databases/repositories (OBIS, GBIF, and NOAA summer bottom trawl surveys) for 69 marine taxa belonging to five different guilds (marine mammals, seabirds, fish, sharks, and crustaceans; Table S4). For Ocean Biodiversity Information System, OBIS (‘*robis*’, https://rdrr.io/cran/robis/man/robis.html; date accessed: 05 July 2021) and the Global Biodiversity Information Facility, GBIF (*‘rgbif’*, https://cran.r-project.org/web/packages/rgbif/index.html; date accessed: 11 August 2021), species records were accessed using their respective R client’s application programming interfaces. The data from Alaska groundfish bottom trawl surveys for the subset of species included in the analyses were downloaded online at https://www.fisheries.noaa.gov/alaska/commercial-fishing/alaska-groundfish-bottom-trawl-survey-data (date accessed; 26 July 2021). All species were further grouped into two marine communities, either as apex (marine mammals, seabirds and sharks) or mesopredators (fish and crustaceans) for biodiversity and species co-occurrence analyses.

Environmental parameters were then extracted for each geographical coordinates from the annual and spatially-thinned species occurrence data and pooled together (2000–2019) to obtain the ranges that were used for environmental profiling during the subsequent pseudo-absence selection. Pseudo-absence selection was conducted by first placing a 100-km radius buffer around the annually-compiled presence points and then using random sampling to select 10,000 pseudo-absences for each year, yielding pooled pseudo-absences equal to 10,000× the number of years with presence records. From these pseudo-absence datasets, 10,000 data points were randomly selected for initial model runs^66^. This was done to select the most parsimonious set of environmental variables important for predicting species-specific potential distributions. For the second round of pseudo-absence selection for final species-specific distribution models, we employed an environmental profiling approach implemented within the ‘mopa’ R package (https://github.com/SantanderMetGroup/mopa) to exclude pixels within the occupied ranges of selected environmental factors and randomly selected 10,000 pseudo-absences from pixels outside of variables’ ranges and the 100-km radius buffer around the presence points for each year^66^.

### Construction of species distribution models

To select the final environmental variables for running the species-specific models, we ran 10 models using different algorithms available and implemented within the ‘biomod2’ R package^67^ with all of the 18 compiled covariates to obtain the averaged variable importance for each environmental factor. We selected the environmental parameters with variable importance greater than the average contribution across each species for the final model runs (Table S5; Fig. S10).

Based on the results of initial model simulations for variable selection, the final species-specific models were developed based on an ensemble model approach of the biomod2 package^67^, using the species-specific presence/pseudo-absence and environmental data over the 20-year period. A total of ten single algorithm models within the biomod2 package were simulated and committee mean ensemble models for each species were computed based on True Skill Statistic (TSS) thresholds (Table S6)^6,33^. The committee mean ensemble model represents the average of binary predictions from selected single algorithm models. The predictive accuracy of ensemble models for each species was further assessed using prevalence-dependent (kappa statistic and AUC) and independent (TSS) metrics which showed high predictive accuracy overall (Table S7). Species-specific spatial distributions were then generated and constrained to eight marine areas where the species were known to exist based on expert knowledge (Table S4). This was done to minimize bias from over-prediction of species ranges prior to implementing habitat range size calculation and biodiversity analyses.

### Species distribution range size changes under contrasting sea ice conditions

To identify periods of low and high summer sea ice concentration (SSIC) at each of the eight CBMP marine areas, we computed annual anomalies relative to the 30-year average in each region (1982–2011) (Fig. S11). As each Arctic area highlighted different numbers of years of high and low sea ice periods, all respective years commonly classified into high and low summer sea ice concentration were pooled together and served as the basis for regional analyses of biodiversity patterns and potential species co-occurrences under contrasting summer sea ice conditions.

Annual species-specific predictions from the multi-model ensemble for high and low summer sea ice years (Fig. S11) were averaged and transformed into binary predictions. These were then used to compute species-specific habitat range size changes between high and low summer sea ice concentration in the last 20 years (2000–2019). Species-specific habitat range size between high and low summer sea ice were then computed using ‘*BIOMOD_RangeSize*’ function of the biomod2 R package^67^. This function computes the proportion and relative frequency of pixels recording loss, gain, and stable habitats between two time slices. Using these outputs, we then identified species that have shown habitat accrual (i.e., habitat gain > habitat loss) and reduction (i.e., habitat gain < habitat loss) between high and low summer sea ice.

### Biodiversity metrics

From the model-derived species-specific distributions, we computed alpha and beta-diversity metrics. Alpha-diversity (species richness, SR) is computed as the pixel-wise total number of species in each year and sea ice periods. Using the annual species richness, we also mapped and examined the spatial distributions of SR temporal trends over the last 20 years to identify areas that have accrued and lost species over time. In particular, areas of increasing richness of at least one species/decade during the last 20 years, were identified as potential emerging hotspots (areas of species accrual) due to climate-driven species redistribution. Further, a temporal beta-diversity index that measures the dissimilarity of community species composition was also computed using presence-absence data from model-derived binary maps of averaged species distributions across all years of high and low sea ice. The metric was computed using the ‘*beta.pair*’ function of the betapart R package^68^, expressed in terms of the Sorensen dissimilarity index. This analysis was done to examine the differences in the community species composition and its components (i.e., turnover and nestedness). Here, the turn-over component of beta-diversity captures the change in species composition due to species replacement while nestedness accounts for composition dissimilarity due to species loss without replacement^68^.

### Species co-occurrence

To examine region-wise numbers of potential species co-occurrences during the 20-year period and between high and low summer sea ice periods, we calculated significant co-occurrences between species pairs at each CBMP area using the ‘*coocc*ur’ function of the ‘cooccur-package’ in R^69^. This function applies the probabilistic model of species co-occurrence^70^ to a species pool distributed among a set of sampling sites (pixels) within each CBMP area to test for statistically significant pairwise patterns of species co-occurrence. It calculates the observed and expected co-occurrence frequencies between each species pair. It returns the probability that two species co-occur more or less frequently than expected if the two species were distributed independently among sites. Potential species associations are further classified as positive (co-occurring pairs), negative (never co-occurring pairs) or random (truly random species pairs).

### Arctic climate and productivity hotspots

To identify areas of potential climate and productivity hotspots throughout the different Arctic marine areas, we calculated the pixel–wise Mann–Kendall trends^71^ to identify areas with large climate and productivity changes derived from the 20-year annual anomalies of summer sea surface temperature (SSST), sea ice concentration (SSIC), chlorophyll-*a* concentration (SCHL) and zooplankton biomass (SZOOC). The approach was earlier implemented to identify marine climate refugia (areas with stable climate over time)^72,73^ and pelagic habitat hotspots^74^. For this work, we used the method to detect climate and productivity hotspots, defined as areas of overlap for cells recording large decreases in SSIC and large increases in SSST, SCHL, and SZOOC over the 20-year period.

## Supplementary Information

Supplementary Information.

## Data Availability

Occurrence records for 69 marine species are publicly available through R clients for the Ocean Biodiversity Information System (OBIS; ‘*robis*’, https://rdrr.io/cran/robis/man/robis.html) and Global Biodiversity Information Facility (GBIF) application programming interfaces (*‘rgbif’*, https://cran.r-project.org/web/packages/rgbif/index.html), and repository of National Oceanic and Atmospheric Administration (NOAA; https://www.fisheries.noaa.gov/alaska/commercial-fishing/alaska-groundfish-bottom-trawl-survey-data). Distance to the nearest coast is available from the Ocean Color website of the National Aeronautics and Space Administration (NASA) (https://oceancolor.gsfc.nasa.gov/docs/distfromcoast/). Daily data of sea ice and sea surface temperature were also publicly available and downloaded from the NOAA Physical Sciences Laboratory (PSL; https://psl.noaa.gov/data/gridded/data.noaa.oisst.v2.highres.html). The daily data of zooplankton and epinekton biomass, monthly chlorophyll-a concentration and salinity from global biogeochemical models were downloaded via registered file transfer protocol from Copernicus Marine Environment Monitoring Service (https://resources.marine.copernicus.eu/).
