# Publisher Correction: Pan-Arctic marine biodiversity and species co-occurrence patterns under recent climate

**DOI:** 10.1038/s41598-023-31756-9

**Published:** 2023-03-20

**Authors:** Irene D. Alabia, Jorge García Molinos, Takafumi Hirata, Franz J. Mueter, Carmen L. David

**Affiliations:** 1 Arctic Research Center, Hokkaido University, N21 W11, Kita-Ku, Sapporo, Hokkaido 001-0021 Japan; 2 College of Fisheries and Ocean Sciences, University of Alaska Fairbanks, 17101 Point Lena Loop Rd, 315 Lena Point Bldg, Juneau, AK 99801-8344 USA; 3 Wageningen University and Research, Wageningen, 6708 PB The Netherlands

Correction to: *Scientific Reports* 10.1038/s41598-023-30943-y, published online 11 March 2023

The original version of this Article contained a typographical error.

Figure 6 did not display correctly.

The original Figure 6 and accompanying legend appears below.

**Figure 6:**
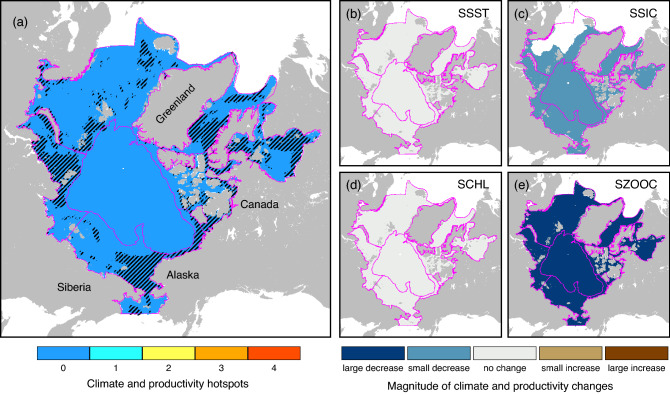
(**a**) Climate and productivity hotspots in Arctic marine areas defined as areas of overlap of two or more large changes in summer (**b**) sea surface temperature (0.21 °C ≤ SSST ≤ 0.76 °C), (**c**) sea ice concentration (− 2% ≥ SSIC ≥ − 76%), (**d**) chlorophyll-a concentration (0.26 mg·m^−3^ ≤ SCHL ≤ 0.73 mg·m^−3^), and (**e**) zooplankton biomass (0.21 g·m^−2^ ≤ SZOOC ≤ 0.75 g·m^−2^). Hatched areas in (**a**) correspond to areas of species accrual (defined as areas with species gains ≥ 1 species/decade) over the entire study period. The maps were created using GMT 6.3.0 (https://docs.generic-mapping-tools.org/6.3/gmt.html).

The original Article has been corrected.

