# Supplementary material for: Pan-Arctic marine biodiversity and species co-occurrence patterns under recent climate
Source: Sci Rep. 2023 Mar 11;13:4076. doi: 10.1038/s41598-023-30943-y (PMC10008629; doi:10.1038/s41598-023-30943-y)
Supplement: Supplementary file 1 — Supplementary Information. [file 41598_2023_30943_MOESM1_ESM.pdf]

Pan-Arctic marine biodiversity and species co-occurrence patterns under recent climate

Irene D. Alabia[1*], Jorge Garcia Molinos[1], Takafumi Hirata[1],
Franz J. Mueter[2], and Carmen L. David[3]

[1]Arctic Research Center, Hokkaido University, N21 W11, Kita-ku, Sapporo, 001-0021 Hokkaido, Japan
[2]College of Fisheries and Ocean Sciences, University of Alaska Fairbanks, 17101 Point Lena Loop Rd, 315 Lena Point bldg. Juneau, AK 99801-8344
[3]Wageningen University and Research, 6708 PB Wageningen, the Netherlands
*irenealabia@arc.hokudai.ac.jp

Supplementary Materials

List of Tables

Table S1. Frequency of sites identified as areas of species accrual (species gains ≥ 1 species/decade) and percentage relative to the total number of pixels at each Arctic areas (values in parentheses) between 2000 and 2019.

Table S2. Pearson's product moment correlation (*R*) between annual averaged species richness (SR) and co-occurrences (positive, negative and random species pair co-occurrences) across the eight CBMP marine areas from 2000-2019.

Table S3. List of environmental data between 2000 and 2019 used for mapping species distributions. Unshaded and shaded parameters were derived from satellite data and biogeochemical model outputs, respectively.

Table S4. List of marine Arctic and sub-arctic taxa used for constructing species distribution models and implementing biodiversity analyses.

Table S5. Variable importance of environmental parameters and values in bold correspond to environmental factors used for constructing the final species distribution models for each taxon.

Table S6. Species-specific model performance metrics based on the True Skill Statistic (TSS) for single algorithm models. Species-specific ensemble models using the committee mean were generated from individual models with TSS higher than the threshold (bold values).

Table S7. Predictive performance (model accuracy) of species-specific committee mean ensemble models assessed based on prevalence-dependent (kappa statistic and Area under the ROC curve, AUC) and prevalence-independent (True Skill Statistics, TSS) metrics.

List of Figures

Figure S1. Annual time-series of area-averaged species richness, with the respective standard deviation (±1 SD) and temporal trends for (a) Pacific Arctic, (b) Beaufort, (c) Arctic Archipelago, (d) Arctic Basin, (e) Atlantic Arctic, (f) Kara-Laptev, (g) Hudson Complex, and (h) Davis-Baffin between 2000 and 2019. The maps were created using GMT 6.3.0 (https://docs.generic-mapping-tools.org/6.3/gmt.html).

Figure S2. Spatial distributions of species richness for apex predators from 2000-2019. The maps were created using GMT 6.3.0 (https://docs.generic-mapping-tools.org/6.3/gmt.html).

Figure S3. Spatial distributions of species richness for mesopredators from 2000-2019. The maps were created using GMT 6.3.0 (https://docs.generic-mapping-tools.org/6.3/gmt.html).

Figure S4. Spatial distributions of species richness for all species from 2000-2019. . The maps were created using GMT 6.3.0 (https://docs.generic-mapping-tools.org/6.3/gmt.html).

Figure S5. Turnover (left panels) and nestedness (right panels) components of beta-diversity between high and low sea ice for (a) apex predators, (b) mesopredators and (c) all species. . The maps were created using GMT 6.3.0 (https://docs.generic-mapping-tools.org/6.3/gmt.html).

Figure S6. Spatial distributions of original and unthinned records (42,579) for species with the most number of occurrences (northern fulmar, *Fulmarus glacialis*) between 2000 and 2019. The maps were created using GMT 6.3.0 (https://docs.generic-mapping-tools.org/6.3/gmt.html).

Figure S7. Spatial distributions of thinned records at 25-km cell size (11,722) for species with the most number of occurrences (northern fulmar, *Fulmarus glacialis*) between 2000 and 2019. The maps were created using GMT 6.3.0 (https://docs.generic-mapping-tools.org/6.3/gmt.html).

Figure S8. Spatial distributions of thinned records at 50-km cell size (6,289) for species with the most number of occurrences (northern fulmar, *Fulmarus glacialis*) between 2000 and 2019. The maps were created using GMT 6.3.0 (https://docs.generic-mapping-tools.org/6.3/gmt.html).

Figure S9. Spatial distributions of thinned records at 100-km cell size (3,338) for species with the most number of occurrences (northern fulmar, *Fulmarus glacialis*) between 2000 and 2019. The maps were created using GMT 6.3.0 (https://docs.generic-mapping-tools.org/6.3/gmt.html).

Figure S10. Number of species belonging to apex (orange bars) and mesopredators (blue bars) selecting specific environmental parameters for the final species distribution models.

Figure S11. Region-specific annual summer sea ice concentration (colored bars) and summer sea surface temperature (SST; solid lines and circles) anomalies from 2000 to 2019 computed relative to the 30-year average for each parameter (1982-2011).

Table S1. Frequency of sites identified as areas of species accrual (species gains ≥ 1 species/decade) and percentage relative to the total number of pixels at each Arctic areas (values in parentheses) between 2000 and 2019.

**CBMP areas**	**Frequency of biodiversity hotspots**		
	**Apex predators**	**Mesopredators**	**All species**
Pacific Arctic	2400 (31%)	1083 (14%)	3285 (42%)
Beaufort	477 (56%)	290 (34%)	666 (78%)
Arctic Archipelago	1939 (48%)	192 (5%)	2022 (50%)
Arctic Basin	171 (1%)	7 (0.1%)	184 (1%)
Atlantic Arctic	2183 (14%)	1655 (11%)	3513 (23%)
Kara-Laptev	2976 (56%)	600 (11%)	3366 (63%)
Hudson Complex	3432 (72%)	115 (2%)	2875 (60%)
Davis-Baffin	1895 (45%)	1092 (26%)	2294 (55%)

Table S2. Pearson's product moment correlation (*R*) between annual averaged species richness (SR) and co-occurrences (positive, negative and random species pair co-occurrences) across the eight CBMP marine areas from 2000-2019.

CBMP marine areas	Correlation (*R*) between species richness and co-occurrence		
	Positive species co-occurrence	Negative species co-occurrence	Random species co-occurrence
Pacific Arctic	0.36	0.44	0.12
Beaufort	0.69***	0.79***	0.82***
Arctic Archipelago	0.62**	0.25	0.32
Arctic Basin	0.04	0.35	0.53*
Atlantic Arctic	0.15	0.12	0.19
Kara-Laptev	0.50*	0.55*	0.73***
Hudson Complex	0.24	-0.03	0.62**
Davis-Baffin	0.10	0.47*	0.41

Significance levels at *p* less than 0.05 (*), 0.01 (**), or 0.001(***)

Table S3. List of environmental data between 2000 and 2019 used for mapping species distributions. Unshaded and shaded parameters were derived from satellite data and biogeochemical model outputs, respectively.

Environmental parameter	Abbreviation	Description/ Unit	Raw resolution	Source
Winter sea surface temperature	WSST	JFMA mean (°C)	0.25°, daily	AVHRR-OI
Summer sea surface temperature	SSST	MJJA mean (°C)		
Winter sea ice concentration	WSIC	JFMA mean (%)		
Summer seaice concentration	SSIC	MJJA mean (%)		
Distance to the seaice edge	DSIE	15% threshold (m)		
Distance to coast	DTC	km	0.04°	NASA
Depth	Dep	m	0.016°	NOAA
Winter zooplankton	WZOOC	JFMA mean (g/m^2)	0.083°, daily	CMEMS
Summer zooplankton	SZOOC	MJJA mean (g/m^2)		
Autumn zooplankton	AZOOC	SOND mean (g/m2)		
Winter epipelagic nekton	WEN	JFMA mean (g/m^2)		
Summer epipelagic nekton	SEN	MJJA mean (g/m^2)		
Autumn epipelagic nekton	AEN	SOND mean (g/m2)		
Winter surface salinity	WSS	JFMA mean (psu)	0.25°, monthly	
Summer surface salinity	SSS	MJJA mean(psu)		
Winter chlorophyll-a	WCHL	JFMA mean (mg/m^3)		
Summer chlorophyll-a	SCHL	MJJA mean (mg/m^3)		
Autumn chlorophyll-a	ACHL	SOND mean (mg/m^3)		

Table S4. List of marine Arctic and sub-arctic taxa used for constructing species distribution models and implementing biodiversity analyses.

Group	Guild	Scientific name	Common name	Expert range distribution	Reference
Apex predators	Marine mammals	*Balaena mysticetus*	Bowhead whale	Pan-Arctic	https://www.iucnredlist.org/ja/species/2467/50347659
		Balaenoptera acutorostrata	Common minke whale	Pan-Arctic	https://www.iucnredlist.org/ja/species/2474/50348265
		Delphinapterus leucas	Beluga	Pan-Arctic	https://www.iucnredlist.org/ja/species/6335/50352346
		Erignathus barbatus	Bearded seal	Pan-Arctic	https://www.iucnredlist.org/ja/species/8010/45225428
		Eschrichtius robustus	Gray whale	Pan-Arctic	https://www.iucnredlist.org/ja/species/8097/50353881
		Monodon monoceros	Narwhal	Davis-Baffin, Hudson; Arctic archipelago; Atlantic Arctic	https://www.iucnredlist.org/ja/species/13704/50367651
		Odobenus rosmarus	Walrus	Pan-Arctic	https://www.iucnredlist.org/ja/species/15106/45228501
		Phoca largha	Spotted seal	North Pacific; Beaufort	https://www.iucnredlist.org/ja/species/17023/45229806
		Pusa hispida	Ringed seal	Pan-Arctic	https://www.iucnredlist.org/ja/species/41672/45231341
		Ursus maritimus	Polar bear	Pan-Arctic	https://www.iucnredlist.org/ja/species/22823/14871490
	Seabirds	*Aethia cristatella*	Crested auklet	North Pacific	https://www.iucnredlist.org/ja/species/22694915/131877037
		Aethia pusilla	Least auklet	North Pacific	https://www.iucnredlist.org/ja/species/22694921/168850125
		Alle alle	Dovekie	Pan-Arctic	https://www.iucnredlist.org/ja/species/22694837/131932114
		Cepphus grylle	Black Guillemot	Pan-Arctic	https://www.iucnredlist.org/ja/species/22694861/132577878
		Fulmarus glacialis	Northern fulmar	Pan-Arctic	https://www.iucnredlist.org/ja/species/22697866/132609419
		Larus hyperboreus	Glaucous Gull	Pan-Arctic	https://www.iucnredlist.org/ja/species/22694343/132544122
		Rissa tridactyla	Black-legged kittiwake	Pan-Arctic	https://www.iucnredlist.org/ja/species/22694497/155617539
		Somateria mollissima	Common eider	Pan-Arctic	https://www.iucnredlist.org/ja/species/22680405/132525971
		Somateria spectabilis	King eider	Pan-Arctic	https://www.iucnredlist.org/ja/species/22680409/132526730
		Uria aalge	Common murre	Atlantic Arctic; Pacific Arctic; Baffin	https://www.iucnredlist.org/ja/species/22694841/132577296
		Uria lomvia	Thick-billed murre	Atlantic Arctic; Pacific Arctic; Baffin	https://www.iucnredlist.org/ja/species/22694847/132066134
	Elasmo-branch	*Lamna ditropis*	Salmon shark	North Pacific	https://www.iucnredlist.org/ja/species/39342/124402990
		Lamna nasus	Porbeagle shark	Atlantic Arctic	https://www.iucnredlist.org/ja/species/11200/500969
		Somniosus microcephalus	Greenland shark	Atlantic Arctic; Davis-Baffin; Hudson; Arctic Archipelago	https://www.iucnredlist.org/ja/species/60213/124452872
		Somniosus pacificus	Pacific sleeper shark	North Pacific	https://www.iucnredlist.org/ja/species/161403/887942
		Squalus acanthias	Spiny dogfish	Davis-Baffin; Atlantic Arctic	https://www.iucnredlist.org/ja/species/91209505/124551959
Meso-predators	Ray-finned fish	*Artediellus atlanticus*	Atlantic hookear sculpin	Pan-arctic	https://www.fishbase.se/summary/Artediellus-atlanticus
		Atheresthes stomias	Arrowtooth flounder	North Pacific	https://www.fishbase.se/summary/Atheresthes-stomias.html
		Boreogadus saida	Arctic cod	Pan-Arctic	https://www.fishbase.se/summary/Boreogadus-saida.html
		Clupea harengus	Atlantic herring	Atlantic Arctic; Davis-Baffin; Hudson	https://www.fishbase.se/summary/Clupea-harengus.html
		Clupea pallasii	Pacific herring	North Pacific; Beaufort	https://www.fishbase.se/summary/Clupea-pallasii.html
		Eleginus gracilis	Saffron cod	Pacific Arctic; Beaufort; Kara-Laptev	https://www.fishbase.se/summary/Eleginus-gracilis.html
		Eutrigla gurnardus	Grey gurnard	Atlantic Arctic	https://www.fishbase.se/summary/Eutrigla-gurnardus.html
		Gadus chalcogrammus	Walleye pollock	North Pacific	https://www.fishbase.se/summary/Gadus-chalcogrammus.html
		Gadus macrocephalus	Pacific cod	Pacific Arctic; Beaufort; Arctic Archipelago; Davis-Baffin	https://www.fishbase.se/summary/Gadus-macrocephalus.html
		Gadus morhua	Atlantic cod	Atlantic Arctic; Davis-Baffin; Hudson; Kara-Laptev	https://www.fishbase.se/summary/Gadus-morhua.html#
		Glyptocephalus zachirus	Rex sole	North Pacific	https://www.fishbase.se/summary/Glyptocephalus-zachirus.html
		Hippoglossoides elassodon	Flathead sole	North Pacific	https://www.fishbase.se/summary/Hippoglossoides-elassodon.html
		Hippoglossoides platessoides	American plaice	Davis-Baffin; Atlantic Arctic	https://www.fishbase.se/summary/Hippoglossoides-platessoides.html
		Hippoglossoides robustus	Bering flounder	Pacific Arctic; Beaufort; Arctic Archipelago	https://www.fishbase.se/summary/Hippoglossoides-robustus.html
		Hippoglossus hippoglossus	Atlantic halibut	Davis-Baffin; Atlantic Arctic	https://www.fishbase.se/summary/Hippoglossus-hippoglossus.html
		Hippoglossus stenolepis	Pacific halibut	North Pacific; Beaufort	https://www.fishbase.se/summary/Hippoglossus-stenolepis.html

Table S4 (continued).

Group	Guild	Scientific name	Common name	Expert range distribution	Reference
Meso-predators	Ray-finned fish	*Lepidopsetta polyxystra*	Northern rock sole	North Pacific	https://www.fishbase.se/summary/Lepidopsetta-polyxystra.html
		Limanda aspera	Yellowfin sole	North Pacific; Beaufort	https://www.fishbase.se/summary/Limanda-aspera.html
		Limanda limanda	Common dab	Atlantic Arctic	https://www.fishbase.se/summary/Limanda-limanda.html
		Limanda proboscidea	Longhead dab	North Pacific; Beaufort	https://www.fishbase.se/summary/Limanda-proboscidea.html
		Mallotus villosus	Capelin	Pan-Arctic	https://www.fishbase.se/summary/Mallotus-villosus.html
		Melanogrammus aeglefinus	Haddock	Davis-Baffin; Atlantic Arctic	https://www.fishbase.se/summary/Melanogrammus-aeglefinus.html
		Merlangius merlangus	Whiting	Atlantic Arctic	https://www.fishbase.se/summary/Merlangius-merlangus.html
		Myoxocephalus polyacanthocephalus	Great sculpin	North Pacific	https://www.fishbase.se/summary/Myoxocephalus-polyacanthocephalus.html
		Myoxocephalus scorpius	Shorthorn sculpin	Atlantic Arctic; Davis-Baffin; Hudson; Pacific Arctic	https://www.fishbase.se/summary/Myoxocephalus-scorpius.html
		Platichthys stellatus	Starry flounder	North Pacific; Beaufort	https://www.fishbase.se/summary/Platichthys-stellatus.html
		Pleuronectes platessa	European plaice	Atlantic Arctic	https://www.fishbase.se/summary/Pleuronectes-platessa.html
		Pleuronectes quadrituberculatus	Alaska plaice	North Pacific	https://www.fishbase.se/summary/Pleuronectes-quadrituberculatus.html
		Pollachius virens	Saithe	Atlantic Arctic; Davis-Baffin; Hudson	https://www.fishbase.se/summary/Pollachius-virens.html
		Reinhardtius hippoglossoides	Greenland halibut	Pan-Arctic	https://www.fishbase.se/summary/Reinhardtius-hippoglossoides.html
		Salvelinus alpinus	Arctic char	Pan-arctic	https://www.fishbase.se/summary/Salvelinus-alpinus.html
		Sebastes mentella	Beaked redfish	Davis-Baffin; Atlantic Arctic	https://www.fishbase.se/summary/Sebastes-mentella.html#
		Sebastes norvegicus	Golden redfish	Atlantic Arctic; Davis-Baffin; Hudson; Kara-Laptev	https://www.fishbase.se/summary/Sebastes-norvegicus.html
		Sprattus Sprattus	European sprat	Atlantic Arctic	https://www.fishbase.se/summary/Sprattus-sprattus.html
		Thaleichthys pacificus	Eulachon	North Pacific	https://www.fishbase.se/summary/Thaleichthys-pacificus.html
		Trisopterus esmarkii	Norway pout	Atlantic Arctic	https://www.fishbase.se/summary/Trisopterus-esmarkii.html
	Crusta-ceans	*Chionoecetes bairdi*	Tanner crab	Pacific Arctic; Davis-Baffin	https://www.sealifebase.ca/summary/Chionoecetes-bairdi.html
		Chionoecetes opilio	Snow crab	Pan-Arctic	https://www.sealifebase.ca/summary/Chionoecetes-opilio.html
		Erimacrus isenbeckii	Horsehair crab	North Pacific	https://www.sealifebase.ca/summary/Erimacrus-isenbeckii.html
		Lithodes aequispinus	Golden king crab	North Pacific	https://www.sealifebase.ca/summary/Lithodes-aequispinus.html
		Pandalus eous	Alaskan pink shrimp	North Pacific	https://www.sealifebase.ca/summary/Pandalus-eous.html
		Paralithodes camtschaticus	Red king crab	Pacific Arctic; Atlantic Arctic; Kara-Laptev	https://www.sealifebase.ca/summary/Paralithodes-camtschaticus.html
		Paralithodes platypus	Blue king crab	North Pacific	https://www.sealifebase.ca/summary/Paralithodes-platypus.html

Table S5. Variable importance of environmental parameters and values in bold correspond to environmental factors used for constructing the final species distribution models for each taxon.

Species	Productivity									Climate and topography									Mean Contribution
	SCHL	ACHL	WCHL	SZOOC	AZOOC	WZOOC	SEN	AEN	WEN	SSIC	WSIC	SSST	WSST	SSAL	WSAL	Dep	DTC	DSIE	
Balaena mysticetus	0.076	**0.341**	**0.170**	**0.125**	**0.278**	0.080	0.065	0.110	0.070	0.070	0.094	**0.130**	0.076	0.075	0.093	**0.160**	0.114	0.116	0.124
Balaenoptera acutorostrata	0.038	0.022	0.023	**0.171**	**0.128**	**0.168**	0.054	0.057	0.029	0.061	**0.126**	**0.245**	**0.426**	0.079	**0.119**	**0.168**	0.098	0.017	0.113
Delphinapterus leucas	0.135	**0.222**	0.128	0.132	**0.228**	0.126	0.145	**0.238**	0.104	0.106	0.095	0.094	0.137	0.090	0.107	0.097	**0.414**	0.100	0.150
Erignathus barbatus	0.063	**0.253**	0.075	0.076	**0.181**	0.091	0.049	0.042	0.066	0.057	0.061	0.102	0.088	0.101	**0.125**	**0.224**	**0.217**	0.082	0.108
Eschrichtius robustus	**0.221**	0.153	0.071	**0.236**	0.150	0.138	0.107	0.086	0.089	0.069	0.079	0.155	0.091	0.114	0.132	**0.558**	0.149	**0.204**	0.156
Monodon monoceros	0.122	0.090	0.168	**0.349**	**0.420**	0.234	**0.261**	0.196	0.210	**0.264**	**0.275**	**0.272**	**0.308**	0.157	0.156	**0.274**	0.222	**0.355**	0.241
Odobenus rosmarus	0.078	0.092	0.041	**0.119**	0.092	0.073	0.086	0.084	0.045	0.091	0.084	**0.131**	**0.128**	**0.168**	0.043	**0.390**	**0.284**	0.084	0.117
Phoca largha	0.176	**0.321**	0.081	**0.434**	0.154	0.183	0.146	**0.222**	0.060	0.101	0.165	0.152	0.164	0.117	0.148	**0.643**	0.106	0.113	0.194
Pusa hispida	0.045	**0.185**	0.097	**0.165**	**0.115**	0.094	0.091	0.064	0.073	0.043	0.092	**0.115**	0.049	**0.170**	0.084	0.088	**0.272**	0.033	0.104
Ursus maritimus	0.101	0.087	0.118	**0.172**	0.124	0.065	0.103	0.068	0.077	0.133	0.094	**0.288**	0.067	**0.159**	**0.166**	0.126	**0.311**	**0.159**	0.196
Aethia cristatella	**0.240**	0.077	0.099	**0.413**	**0.237**	**0.182**	0.068	0.060	0.121	0.049	0.035	0.110	0.097	**0.265**	**0.261**	0.130	0.100	0.066	0.145
Aethia pusilla	**0.305**	0.087	0.105	**0.263**	**0.181**	0.141	**0.158**	0.143	0.077	0.059	0.081	0.122	0.057	**0.278**	**0.314**	0.095	0.136	0.042	0.147
Alle alle	**0.137**	0.046	0.052	0.068	**0.107**	**0.116**	0.072	**0.146**	0.038	0.061	0.090	**0.141**	0.033	0.101	**0.238**	0.096	**0.155**	**0.119**	0.101
Cepphus grylle	0.041	0.032	0.026	**0.092**	0.075	0.054	0.033	0.053	0.019	0.072	0.045	0.041	0.059	0.030	**0.094**	**0.084**	**0.626**	0.022	0.083
Fulmarus glacialis	**0.076**	0.037	**0.108**	**0.085**	**0.065**	0.055	0.045	0.034	0.024	0.049	0.045	**0.124**	0.064	**0.084**	0.048	0.044	**0.147**	0.039	0.065
Larus hyperboreus	**0.099**	0.048	0.056	0.057	**0.116**	0.047	0.053	**0.135**	0.031	0.073	0.049	0.062	0.043	0.046	0.033	0.078	**0.356**	0.026	0.078
Rissa tridactyla	**0.077**	0.042	**0.105**	**0.071**	0.054	0.039	0.016	0.037	0.009	0.055	0.050	**0.082**	0.024	0.049	0.044	0.033	**0.213**	0.029	0.057
Somateria mollissima	0.045	0.028	0.045	0.063	0.045	0.044	0.044	0.036	0.022	0.062	0.066	0.052	0.032	0.020	0.021	**0.097**	**0.553**	0.011	0.071
Somateria spectabilis	**0.088**	**0.096**	0.045	**0.172**	0.065	0.081	0.052	0.035	0.023	0.038	0.032	0.060	0.040	0.071	0.036	**0.233**	**0.282**	0.027	0.082
Uria aalge	**0.069**	0.045	**0.192**	0.056	0.060	0.034	0.021	0.030	0.008	0.038	0.036	**0.189**	**0.059**	0.019	0.019	**0.068**	**0.200**	0.014	0.064
Uria lomvia	**0.204**	0.049	0.047	0.070	**0.081**	**0.105**	0.015	0.049	0.023	**0.081**	0.035	0.042	0.027	**0.086**	0.053	**0.124**	**0.120**	**0.132**	0.075
Lamna ditropis	**0.210**	0.110	**0.296**	**0.291**	**0.190**	0.166	**0.194**	0.141	0.147	**0.210**	0.090	**0.200**	**0.204**	0.141	**0.312**	**0.242**	0.074	0.041	0.181
Lamna nasus	0.129	0.046	0.143	**0.380**	0.160	0.168	**0.225**	0.176	0.192	0.089	**0.270**	**0.510**	**0.376**	**0.241**	0.187	**0.462**	0.199	0.048	0.222
Somniosus microcephalus	0.133	**0.254**	**0.231**	0.197	0.170	**0.267**	0.162	**0.224**	0.169	0.141	**0.391**	**0.229**	**0.266**	0.066	0.074	**0.468**	**0.218**	**0.225**	0.216
Somniosus pacificus	0.105	0.108	**0.397**	**0.295**	0.143	0.134	0.086	0.128	0.076	0.136	0.076	0.150	**0.186**	0.119	**0.269**	**0.219**	0.066	0.052	0.153
Squalus acanthias	0.039	0.039	**0.291**	**0.451**	0.098	0.115	0.074	0.058	0.045	0.117	0.103	0.121	**0.247**	**0.133**	0.106	**0.209**	0.066	0.028	0.130
Artediellus atlanticus	0.126	0.083	**0.135**	**0.259**	0.181	0.207	0.553	0.146	0.209	0.114	0.151	0.325	**0.150**	**0.111**	0.084	**0.170**	0.171	0.295	0.193
Atheresthes stomias	0.049	0.058	**0.113**	**0.406**	0.128	0.072	0.068	0.065	0.056	0.058	0.056	0.153	**0.198**	**0.072**	0.155	**0.118**	0.084	0.009	0.107
Boreogadus saida	0.055	0.039	**0.111**	**0.257**	0.087	0.217	0.081	0.155	0.060	0.043	0.186	0.185	**0.077**	**0.072**	0.066	**0.078**	0.054	0.080	0.106
Clupea harengus	0.030	0.023	**0.089**	**0.117**	0.076	0.062	0.044	0.097	0.069	0.057	0.050	0.185	**0.213**	**0.040**	0.062	**0.113**	0.065	0.011	0.078
Clupea pallasii	0.064	0.068	**0.295**	**0.309**	0.129	0.084	0.061	0.093	0.042	0.126	0.075	0.111	**0.121**	**0.089**	0.152	**0.259**	0.054	0.029	0.120
Eleginus gracilis	0.052	0.112	**0.202**	**0.375**	0.257	0.112	0.166	0.163	0.040	0.059	0.082	0.291	**0.125**	**0.104**	0.123	**0.509**	0.115	0.049	0.163

Table S5 (continued)

**Species**	**Productivity**									**Climate and topography**									Mean Contribution
	SCHL	ACHL	WCHL	SZOOC	AZOOC	WZOOC	SEN	AEN	WEN	SSIC	WSIC	SSST	WSST	SSAL	WSAL	Dep	DTC	DSIE	
Eutrigla gurnardus	0.043	0.023	0.107	**0.151**	0.049	0.040	0.026	0.030	0.030	0.096	0.104	**0.386**	**0.157**	**0.111**	0.085	**0.325**	0.054	**0.159**	0.110
Gadus chalcogrammus	0.037	0.055	**0.166**	**0.581**	**0.149**	0.098	**0.143**	0.111	0.082	0.038	0.039	**0.123**	0.096	0.041	**0.159**	**0.179**	0.057	0.011	0.120
Gadus macrocephalus	0.035	0.043	**0.224**	**0.481**	**0.114**	0.069	**0.139**	0.104	0.062	0.040	0.039	0.106	0.086	0.045	**0.137**	**0.213**	0.051	0.012	0.111
Gadus morhua	0.063	0.045	**0.152**	**0.181**	**0.115**	0.078	0.024	0.077	0.029	0.068	0.048	**0.195**	**0.151**	0.038	**0.118**	**0.239**	0.064	0.025	0.095
Glyptocephalus zachirus	0.046	0.043	**0.228**	**0.326**	0.088	0.086	**0.130**	0.054	0.084	0.083	0.045	0.115	**0.390**	0.057	**0.159**	**0.149**	0.057	0.011	0.119
Hippoglossoides elassodon	0.039	0.043	**0.169**	**0.545**	**0.128**	0.098	**0.176**	0.099	**0.125**	0.049	0.043	0.110	0.099	0.058	**0.139**	**0.188**	0.057	0.008	0.121
Hippoglossoides platessoides	0.057	0.031	**0.203**	**0.159**	0.089	**0.119**	0.050	**0.152**	0.049	0.055	0.057	**0.195**	**0.151**	0.039	**0.120**	**0.258**	0.055	0.037	0.104
Hippoglossoides robustus	0.056	0.053	**0.156**	**0.484**	**0.182**	0.120	**0.205**	0.142	0.125	0.061	0.054	**0.162**	0.087	0.081	0.133	**0.440**	0.090	0.033	0.148
Hippoglossus hippoglossus	0.066	0.077	**0.258**	0.120	0.153	0.163	0.083	0.100	0.117	0.134	0.146	**0.657**	**0.244**	0.087	**0.173**	**0.337**	0.079	0.075	0.171
Hippoglossus stenolepis	0.034	0.054	**0.167**	**0.473**	**0.146**	0.086	0.110	0.074	0.055	0.040	0.075	**0.119**	0.079	0.033	**0.170**	**0.196**	0.083	0.014	0.112
Lepidopsetta polyxystra	0.035	0.042	**0.204**	**0.531**	0.120	0.100	**0.141**	**0.146**	0.073	0.042	0.045	**0.182**	0.071	0.063	**0.171**	**0.279**	0.051	0.025	0.129
Limanda aspera	0.037	0.042	**0.169**	**0.519**	**0.134**	0.111	**0.164**	**0.150**	0.073	0.037	0.048	**0.142**	0.067	0.043	**0.151**	**0.247**	0.040	0.025	0.122
Limanda limanda	0.027	0.029	**0.148**	**0.155**	**0.148**	0.043	0.049	0.044	0.030	**0.185**	0.092	**0.354**	**0.145**	0.072	0.097	**0.347**	0.074	**0.152**	0.122
Limanda proboscidea	0.052	0.057	0.135	**0.501**	**0.191**	0.163	**0.194**	**0.165**	0.060	0.046	0.073	**0.285**	0.095	0.094	**0.191**	**0.499**	0.078	0.058	0.163
Mallotus villosus	0.049	0.032	**0.203**	**0.506**	0.075	**0.170**	0.122	**0.198**	0.092	0.034	0.042	0.066	0.081	0.064	0.113	**0.268**	0.058	0.070	0.125
Melanogrammus aeglefinus	0.053	0.023	0.077	**0.238**	**0.123**	0.055	0.030	0.034	0.033	**0.130**	0.067	**0.137**	**0.170**	**0.107**	**0.166**	**0.231**	0.056	0.054	0.099
Merlangius merlangus	0.025	0.018	**0.152**	**0.220**	0.044	0.044	0.029	0.037	0.039	**0.161**	0.093	**0.433**	0.105	0.049	0.097	**0.308**	0.048	**0.139**	0.113
Myoxocephalus polyacanthocephalus	0.046	0.062	0.103	**0.571**	**0.144**	0.120	**0.135**	0.096	0.063	0.062	0.059	**0.135**	0.079	0.069	**0.138**	**0.382**	0.084	0.014	0.131
Myoxocephalus scorpius	0.043	0.061	**0.134**	**0.208**	0.096	**0.156**	**0.114**	**0.208**	0.045	0.050	0.032	**0.141**	0.044	0.056	0.044	**0.322**	**0.117**	0.050	0.107
Platichthys stellatus	0.042	0.072	**0.404**	**0.396**	0.102	0.086	**0.133**	0.075	0.049	0.050	0.069	**0.147**	0.057	0.051	**0.194**	**0.309**	0.077	0.028	0.130
Pleuronectes platessa	0.027	0.019	**0.124**	**0.169**	0.048	0.064	0.032	0.077	0.040	**0.105**	0.086	**0.277**	**0.104**	0.077	0.056	**0.327**	0.065	**0.118**	0.101
Pleuronectes quadrituberculatus	0.031	0.048	**0.201**	**0.589**	**0.135**	0.100	**0.140**	0.127	0.091	0.037	0.042	**0.213**	0.079	0.050	**0.157**	**0.293**	0.066	0.032	0.135
Pollachius virens	0.043	0.034	0.080	**0.177**	0.104	0.057	0.033	0.042	0.034	0.071	**0.109**	**0.282**	**0.169**	0.058	0.104	**0.361**	0.071	0.049	0.104
Reinhardtius hippoglossoides	**0.132**	0.068	**0.194**	**0.211**	0.092	**0.193**	**0.155**	0.034	0.093	0.040	**0.151**	0.100	**0.139**	0.057	0.097	**0.172**	0.066	0.089	0.116
Salvelinus alpinus	**0.273**	0.063	**0.125**	**0.234**	0.300	**0.316**	**0.367**	0.268	0.252	0.219	**0.199**	0.223	**0.348**	0.165	0.063	**0.149**	0.651	0.110	0.240
Sebastes mentella	**0.105**	0.108	**0.173**	**0.326**	0.156	**0.202**	**0.182**	0.250	0.132	0.078	**0.196**	0.394	**0.250**	0.094	0.130	**0.343**	0.074	0.197	0.188
Sebastes norvegicus	**0.241**	0.076	**0.148**	**0.355**	0.109	**0.252**	**0.071**	0.275	0.130	0.141	**0.217**	0.176	**0.419**	0.090	0.150	**0.259**	0.210	0.157	0.193
Sprattus sprattus	**0.033**	0.022	**0.174**	**0.297**	0.062	**0.057**	**0.038**	0.082	0.038	0.144	**0.143**	0.303	**0.096**	0.110	0.064	**0.351**	0.037	0.144	0.122
Thaleichthys pacificus	**0.041**	0.078	**0.285**	**0.516**	0.138	**0.105**	**0.108**	0.159	0.076	0.099	**0.094**	0.133	**0.260**	0.108	0.180	**0.286**	0.082	0.023	0.154
Trisopterus esmarkii	**0.037**	0.020	**0.117**	**0.204**	0.083	**0.053**	**0.038**	0.059	0.037	0.085	**0.136**	0.223	**0.158**	0.099	0.123	**0.283**	0.094	0.172	0.112
Chionoecetes bairdi	**0.042**	0.052	**0.098**	**0.515**	0.089	**0.083**	**0.149**	0.090	0.084	0.069	**0.055**	0.156	**0.207**	0.074	0.142	**0.298**	0.066	0.015	0.127
Chionoecetes opilio	**0.058**	0.050	**0.088**	**0.546**	0.193	**0.100**	**0.152**	0.143	0.050	0.044	**0.041**	0.236	**0.072**	0.070	0.106	**0.308**	0.079	0.021	0.131
Erimacrus isenbeckii	**0.111**	0.104	**0.573**	**0.267**	0.182	**0.167**	**0.189**	0.127	0.133	0.313	**0.135**	0.199	**0.210**	0.176	0.227	**0.281**	0.109	0.137	0.202
Lithodes aequispinus	**0.083**	0.090	**0.403**	**0.185**	0.275	**0.104**	**0.134**	0.188	0.154	0.105	**0.183**	0.260	**0.437**	0.194	0.325	**0.257**	0.095	0.060	0.196
Pandalus eous	**0.116**	0.106	**0.112**	**0.418**	0.143	**0.090**	**0.083**	0.064	0.047	0.044	**0.047**	0.168	**0.135**	0.056	0.156	**0.424**	0.085	0.091	0.132
Paralithodes camtschaticus	**0.041**	0.046	**0.269**	**0.469**	0.158	**0.132**	**0.120**	0.112	0.082	0.055	**0.052**	0.179	**0.078**	0.065	0.139	**0.277**	0.078	0.027	0.132
Paralithodes platypus	**0.246**	0.134	**0.146**	**0.435**	0.205	**0.156**	**0.209**	0.073	0.101	0.280	**0.124**	0.160	**0.123**	0.225	0.263	**0.316**	0.129	0.222	0.197

Table S6. Species-specific model performance metrics based on the True Skill Statistic (TSS) for single algorithm models. Species-specific ensemble models using the committee mean were generated from individual models with TSS higher than the threshold (bold values).

Guild	Species	GAM	GLM	GBM	ANN	RF	MaxEnt	SRE	MARS	FDA	CTA	cutoff
Marine mammal	*Balaena mysticetus*	**0.844**	0.737	0.832	0.772	**0.884**	0.568	0.680	0.821	0.719	**0.856**	0.835
	Balaenoptera acutorostrata	**0.915**	**0.858**	**0.890**	0.906	**0.917**	**0.794**	**0.671**	**0.892**	**0.882**	**0.891**	0.902
	Delphinapterus leucas	0.830	0.807	**0.848**	0.815	**0.875**	0.761	0.689	0.816	0.779	0.835	0.837
	Erignathus barbatus	0.793	0.731	**0.833**	0.719	**0.856**	0.728	0.605	**0.802**	0.763	0.740	0.798
	Eschrichtius robustus	0.907	**0.926**	0.897	0.003	0.905	0.912	0.788	**0.915**	0.874	0.882	0.910
	Monodon monoceros	0.440	0.558	**0.860**	0.144	**0.894**	0.599	0.640	**0.850**	**0.810**	0.787	0.807
	Odobenus rosmarus	0.848	0.764	**0.891**	**0.874**	**0.899**	0.743	0.574	0.836	0.805	0.820	0.861
	Phoca largha	0.784	**0.896**	**0.871**	0.851	**0.908**	0.784	0.651	0.850	0.770	0.803	0.863
	Pusa hispida	0.765	0.614	**0.785**	0.711	**0.809**	0.714	0.622	**0.769**	0.703	**0.769**	0.769
	Ursus maritimus	**0.846**	0.699	0.801	0.793	**0.860**	0.721	0.657	0.806	0.793	0.768	0.817
Seabirds	*Aethia cristatella*	**0.890**	0.816	**0.900**	0.867	**0.898**	0.830	0.752	**0.898**	0.872	0.874	0.889
	Aethia pusilla	**0.895**	0.865	0.876	**0.910**	**0.902**	0.768	0.732	**0.908**	0.851	0.867	0.893
	Alle alle	0.886	0.783	0.881	0.000	**0.948**	0.852	0.618	0.884	0.840	0.889	0.890
	Cepphus grylle	0.851	0.831	**0.879**	0.846	**0.900**	0.804	0.672	0.852	0.851	0.863	0.866
	Fulmarus glacialis	0.872	0.798	0.885	0.885	**0.927**	0.758	0.668	0.864	0.857	0.884	0.886
	Larus hyperboreus	0.836	0.792	**0.872**	**0.876**	**0.888**	0.747	0.606	0.841	0.815	**0.872**	0.864
	Rissa tridactyla	0.852	0.822	**0.872**	0.863	**0.898**	0.809	0.673	0.845	0.841	**0.867**	0.866
	Somateria mollissima	0.904	0.906	**0.910**	0.906	**0.907**	0.838	0.749	**0.907**	0.897	**0.907**	0.907
	Somateria spectabilis	0.875	0.864	**0.897**	**0.904**	**0.919**	0.797	0.749	0.885	0.890	0.889	0.897
	Uria aalge	0.926	0.909	**0.935**	0.920	**0.940**	0.835	0.751	0.925	0.895	0.917	0.927
	Uria lomvia	0.862	0.791	0.862	0.800	**0.922**	0.826	0.669	0.863	0.843	0.858	0.868
Elasmobranch	*Lamna ditropis*	0.860	0.965	**0.977**	0.962	**0.986**	0.942	0.626	**0.990**	0.968	0.959	0.975
	Lamna nasus	0.569	**0.987**	0.843	0.704	**0.956**	**0.992**	0.426	0.567	0.872	0.788	0.906
	Somniosus microcephalus	0.568	0.592	**0.920**	0.725	**0.950**	**0.903**	0.708	0.762	**0.933**	0.722	0.866
	Somniosus pacificus	0.970	0.927	0.969	**0.976**	**0.975**	0.953	0.772	**0.987**	0.914	0.944	0.972
	Squalus acanthias	**0.972**	0.952	0.966	**0.972**	**0.978**	0.884	0.771	0.965	0.927	0.943	0.968
Ray-finned fish	*Artediellus atlanticus*	**0.879**	0.834	**0.879**	0.868	**0.902**	0.851	0.513	0.867	0.807	0.834	0.874
	Atheresthes stomias	**0.982**	0.922	0.977	0.976	**0.989**	0.955	0.800	**0.982**	0.943	0.969	0.979
	Boreogadus saida	0.847	0.793	**0.878**	0.868	**0.919**	0.805	0.744	0.853	0.838	**0.881**	0.874
	Clupea harengus	0.963	0.924	**0.969**	**0.971**	**0.979**	0.823	0.757	0.951	0.917	**0.973**	0.968
	Clupea pallasii	**0.964**	0.953	**0.969**	0.957	**0.969**	0.919	0.789	**0.971**	0.954	0.948	0.964
	Eleginus gracilis	**0.961**	0.939	0.951	**0.964**	0.952	0.864	0.759	**0.962**	0.913	0.912	0.955
	Eutrigla gurnardus	0.990	**0.998**	0.993	**0.996**	**0.997**	0.989	0.808	**0.999**	0.986	0.984	0.996
	Gadus chalcogrammus	**0.966**	0.915	0.955	0.960	**0.977**	0.870	0.773	0.963	0.931	**0.964**	0.964
	Gadus macrocephalus	**0.975**	0.917	0.958	0.961	**0.976**	0.916	0.814	**0.971**	0.932	0.964	0.968
	Gadus morhua	0.909	0.836	**0.927**	0.896	**0.955**	0.807	0.730	0.890	0.792	**0.919**	0.916
	Glyptocephalus zachirus	**0.981**	0.939	0.972	**0.977**	**0.977**	0.949	0.791	**0.983**	0.949	0.964	0.976

Table **S6** (continued)

Guild	Species	GAM	GLM	GBM	ANN	RF	MaxEnt	SRE	MARS	FDA	CTA	cutoff
Ray-finned fish	*Hippoglossoides elassodon*	**0.987**	0.925	0.975	0.979	**0.986**	0.867	0.789	**0.981**	0.951	0.971	0.980
	Hippoglossoides platessoides	0.903	0.766	**0.921**	**0.917**	**0.958**	0.677	0.649	0.876	0.839	**0.918**	0.916
	Hippoglossoides robustus	**0.954**	0.920	**0.957**	0.953	**0.960**	0.849	0.809	**0.962**	0.887	0.940	0.954
	Hippoglossus hippoglossus	**0.944**	0.931	0.942	**0.946**	**0.960**	0.810	0.731	0.921	0.861	0.939	0.944
	Hippoglossus stenolepis	**0.982**	0.910	0.969	0.966	0.983	0.909	0.765	**0.979**	0.940	0.965	0.974
	Lepidopsetta polyxystra	**0.990**	0.934	0.973	**0.983**	**0.987**	0.940	0.764	**0.984**	0.961	0.967	0.981
	Limanda aspera	**0.983**	0.913	0.976	0.975	**0.986**	0.948	0.784	0.977	0.946	0.971	0.978
	Limanda limanda	0.977	0.991	**0.994**	**0.994**	**0.995**	0.919	0.773	**0.995**	0.978	0.986	0.993
	Limanda proboscidea	**0.973**	0.942	**0.964**	0.962	0.953	0.831	0.772	**0.973**	0.924	0.954	0.963
	Mallotus villosus	0.850	0.783	0.870	0.866	**0.918**	0.784	0.754	0.844	0.800	**0.895**	0.874
	Melanogrammus aeglefinus	0.959	0.958	0.969	**0.975**	**0.981**	0.892	0.804	0.965	0.911	0.968	0.970
	Merlangius merlangus	**0.995**	**0.995**	0.992	**0.996**	**0.996**	0.969	0.851	**0.997**	0.989	0.987	0.995
	Myoxocephalus polyacanthocephalus	**0.980**	0.928	0.973	**0.978**	**0.980**	0.887	0.805	**0.980**	0.951	0.964	0.976
	Myoxocephalus scorpius	0.882	0.872	**0.919**	**0.903**	**0.942**	0.651	0.705	0.881	0.813	0.874	0.900
	Platichthys stellatus	**0.986**	0.941	0.979	0.976	**0.988**	0.882	0.731	**0.987**	0.935	0.973	0.982
	Pleuronectes platessa	**0.992**	0.990	**0.991**	0.988	**0.995**	0.907	0.767	**0.991**	0.975	0.981	0.991
	Pleuronectes quadrituberculatus	**0.982**	0.931	0.979	**0.980**	**0.991**	0.943	0.752	**0.982**	0.954	0.968	0.980
	Pollachius virens	0.975	0.972	**0.990**	0.975	**0.991**	0.860	0.891	0.974	0.934	**0.987**	0.982
	Reinhardtius hippoglossoides	0.878	0.713	0.885	**0.893**	**0.942**	0.844	0.754	0.852	0.789	**0.898**	0.891
	Salvelinus alpinus	0.197	**0.877**	0.786	**0.895**	0.600	**0.867**	0.581	0.573	0.684	0.696	0.801
	Sebastes mentella	**0.893**	0.745	**0.904**	0.076	**0.915**	0.814	0.705	**0.913**	0.784	0.822	0.877
	Sebastes norvegicus	0.900	0.716	**0.915**	**0.911**	**0.911**	0.789	0.756	**0.903**	0.803	0.863	0.901
	Sprattus sprattus	**0.999**	**0.998**	**0.999**	0.996	**0.999**	0.943	0.785	**0.998**	0.990	0.994	0.998
	Thaleichthys pacificus	0.985	0.974	**0.990**	0.977	**0.995**	0.984	0.753	**0.992**	0.947	0.974	0.987
	Trisopterus esmarkii	0.988	0.987	**0.991**	**0.991**	**0.993**	0.974	0.792	**0.993**	0.976	0.989	0.991
Crustaceans	*Chionoecetes bairdi*	**0.983**	0.932	0.978	0.975	**0.988**	0.940	0.776	**0.982**	0.954	0.974	0.980
	Chionoecetes opilio	**0.947**	0.903	0.938	**0.956**	**0.950**	0.889	0.821	0.940	0.894	0.936	0.945
	Erimacrus isenbeckii	0.970	0.974	**0.979**	**0.994**	**0.985**	0.897	0.716	0.971	0.930	0.936	0.979
	Lithodes aequispinus	0.876	0.950	0.969	**0.984**	**0.995**	0.943	0.860	**0.984**	0.959	0.958	0.975
	Pandalus eous	**0.984**	0.913	0.973	0.928	**0.987**	0.950	0.789	**0.985**	0.941	0.966	0.974
	Paralithodes camtschaticus	**0.974**	0.929	**0.975**	0.972	**0.979**	0.909	0.759	**0.973**	0.912	0.967	0.973
	Paralithodes platypus	0.805	0.867	**0.948**	0.830	0.858	0.838	0.726	**0.905**	0.869	0.874	0.887

Table S7. Predictive performance (model accuracy) of species-specific committee mean ensemble models assessed based on prevalence-dependent (kappa statistic and Area under the ROC curve, AUC) and prevalence-independent (True Skill Statistics, TSS) metrics.

Species	Common name	Models' predictive accuracy		
		Prevalence-dependent		Prevalence-independent
		kappa	AUC	TSS
Balaena mysticetus	Bowhead whale	0.689	0.985	0.934
Balaenoptera acutorostrata	Common minke whale	0.933	0.990	0.939
Delphinapterus leucas	Beluga	0.677	0.980	0.929
Erignathus barbatus	Bearded seal	0.604	0.970	0.892
Eschrichtius robustus	Gray whale	0.441	0.984	0.936
Monodon monoceros	Narwhal	0.420	0.993	0.948
Odobenus rosmarus	Walrus	0.746	0.985	0.934
Phoca largha	Spotted seal	0.335	0.987	0.936
Pusa hispida	Ringed seal	0.609	0.967	0.860
Ursus maritimus	Polar bear	0.611	0.975	0.895
Aethia cristatella	Crested auklet	0.822	0.989	0.946
Aethia pusilla	Least auklet	0.820	0.990	0.954
Alle alle	Dovekie	0.938	0.989	0.978
Cepphus grylle	Black Guillemot	0.907	0.984	0.924
Fulmarus glacialis	Northern fulmar	0.967	0.985	0.971
Larus hyperboreus	Glaucous Gull	0.875	0.981	0.910
Rissa tridactyla	Black-legged kittiwake	0.918	0.983	0.919
Somateria mollissima	Common eider	0.853	0.951	0.902
Somateria spectabilis	King eider	0.835	0.978	0.934
Uria aalge	Common murre	0.947	0.988	0.953
Uria lomvia	Thick-billed murre	0.946	0.985	0.970
Lamna ditropis	Salmon shark	0.929	0.999	0.995
Lamna nasus	Porbeagle shark	0.429	0.997	0.993
Somniosus microcephalus	Greenland shark	0.756	0.991	0.962
Somniosus pacificus	Pacific sleeper shark	0.931	0.997	0.990
Squalus acanthias	Spiny dogfish	0.972	0.997	0.986
Artediellus atlanticus	Atlantic hookear sculpin	0.540	0.980	0.939
Atheresthes stomias	Arrowtooth flounder	0.990	0.999	0.991
Boreogadus saida	Arctic cod	0.876	0.982	0.923
Clupea harengus	Atlantic herring	0.982	0.997	0.983
Clupea pallasii	Pacific herring	0.950	0.996	0.984
Eleginus gracilis	Saffron cod	0.867	0.995	0.972
Eutrigla gurnardus	Grey gurnard	0.997	1.000	0.999
Gadus chalcogrammus	Walleye pollock	0.983	0.996	0.983
Gadus macrocephalus	Pacific cod	0.983	0.997	0.981
Gadus morhua	Atlantic cod	0.951	0.994	0.957
Glyptocephalus zachirus	Rex sole	0.986	0.999	0.989

Table S7 (continued)

Species	Common name	Models' predictive accuracy		
		Prevalence-dependent		Prevalence-independent
		**kappa**	**AUC**	**TSS**
Hippoglossoides elassodon	Flathead sole	0.986	0.997	0.989
Hippoglossoides platessoides	American plaice	0.953	0.994	0.958
Hippoglossoides robustus	Bering flounder	0.939	0.993	0.974
Hippoglossus hippoglossus	Atlantic halibut	0.888	0.993	0.976
Hippoglossus stenolepis	Pacific halibut	0.981	0.997	0.990
Lepidopsetta polyxystra	Northern rock sole	0.991	0.999	0.992
Limanda aspera	Yellowfin sole	0.988	0.998	0.992
Limanda limanda	Common dab	0.996	1.000	0.997
Limanda proboscidea	Longhead dab	0.949	0.996	0.986
Mallotus villosus	Capelin	0.928	0.986	0.953
Melanogrammus aeglefinus	Haddock	0.985	0.997	0.987
Merlangius merlangus	Whiting	0.996	0.999	0.998
Myoxocephalus polyacanthocephalus	Great sculpin	0.974	0.997	0.985
Myoxocephalus scorpius	Shorthorn sculpin	0.863	0.987	0.948
Platichthys stellatus	Starry flounder	0.982	0.999	0.992
Pleuronectes platessa	European plaice	0.996	0.999	0.997
Pleuronectes quadrituberculatus	Alaska plaice	0.987	0.999	0.992
Pollachius virens	Saithe	0.960	0.997	0.987
Reinhardtius hippoglossoides	Greenland halibut	0.947	0.993	0.952
Salvelinus alpinus	Arctic char	0.036	0.962	0.925
Sebastes mentella	Beaked redfish	0.816	0.988	0.949
Sebastes norvegicus	Golden redfish	0.788	0.989	0.938
Sprattus sprattus	European sprat	0.996	1.000	0.998
Thaleichthys pacificus	Eulachon	0.977	0.999	0.996
Trisopterus esmarkii	Norway pout	0.992	0.999	0.992
Chionoecetes bairdi	Tanner crab	0.988	0.998	0.993
Chionoecetes opilio	Snow crab	0.962	0.994	0.971
Erimacrus isenbeckii	Horsehair crab	0.895	0.998	0.997
Lithodes aequispinus	Golden king crab	0.954	0.999	0.993
Pandalus eous	Alaskan pink shrimp	0.981	0.999	0.993
Paralithodes camtschaticus	Red king crab	0.963	0.997	0.987
Paralithodes platypus	Blue king crab	0.522	0.992	0.973

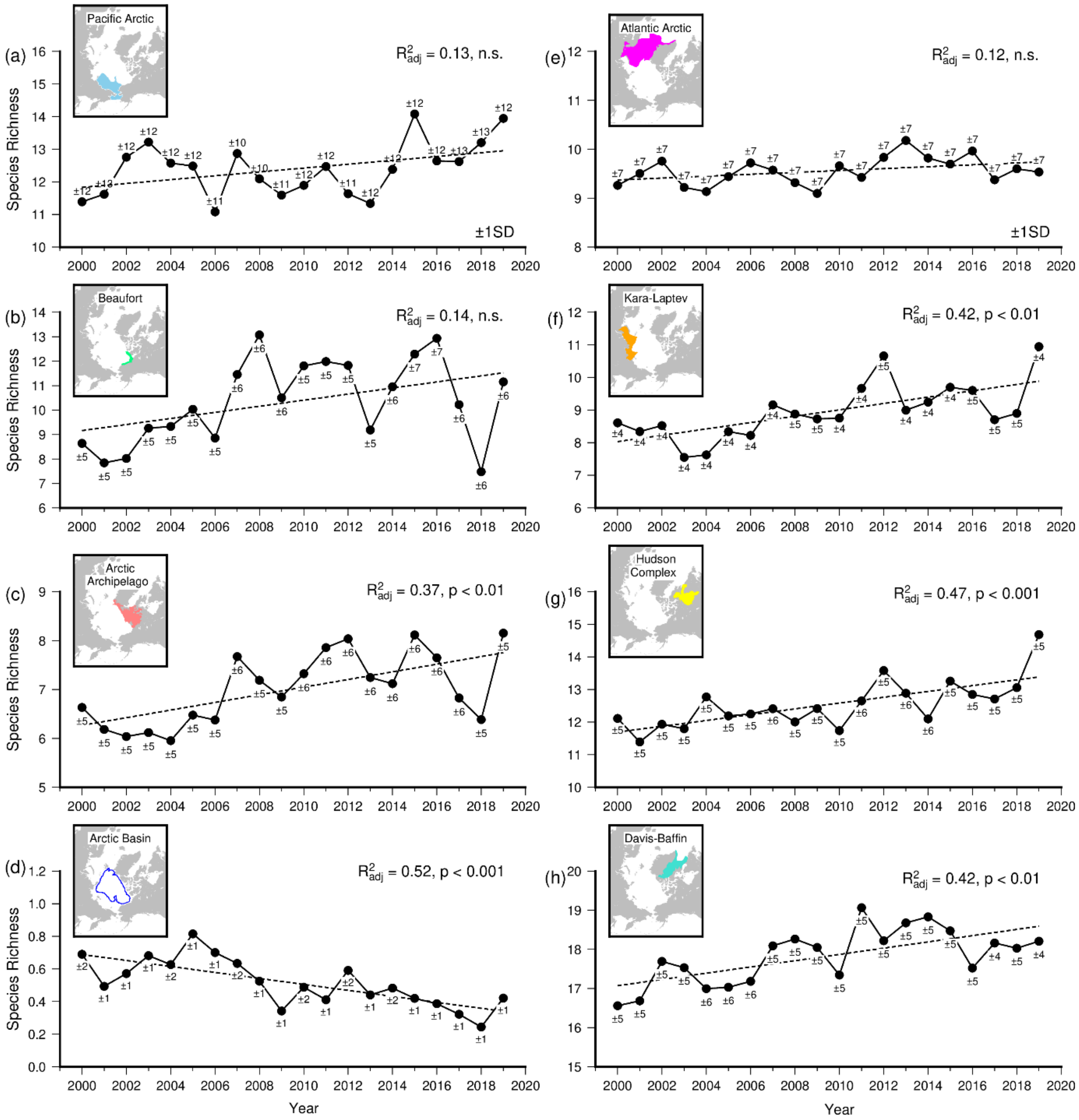

Figure S1. Annual time-series of area-averaged species richness, with the respective standard deviation (±1 SD) and temporal trends for (a) Pacific Arctic, (b) Beaufort, (c) Arctic Archipelago, (d) Arctic Basin, (e) Atlantic Arctic, (f) Kara-Laptev, (g) Hudson Complex, and (h) Davis-Baffin between 2000 and 2019. The maps were created using GMT 6.3.0 (https://docs.generic-mapping-tools.org/6.3/gmt.html).

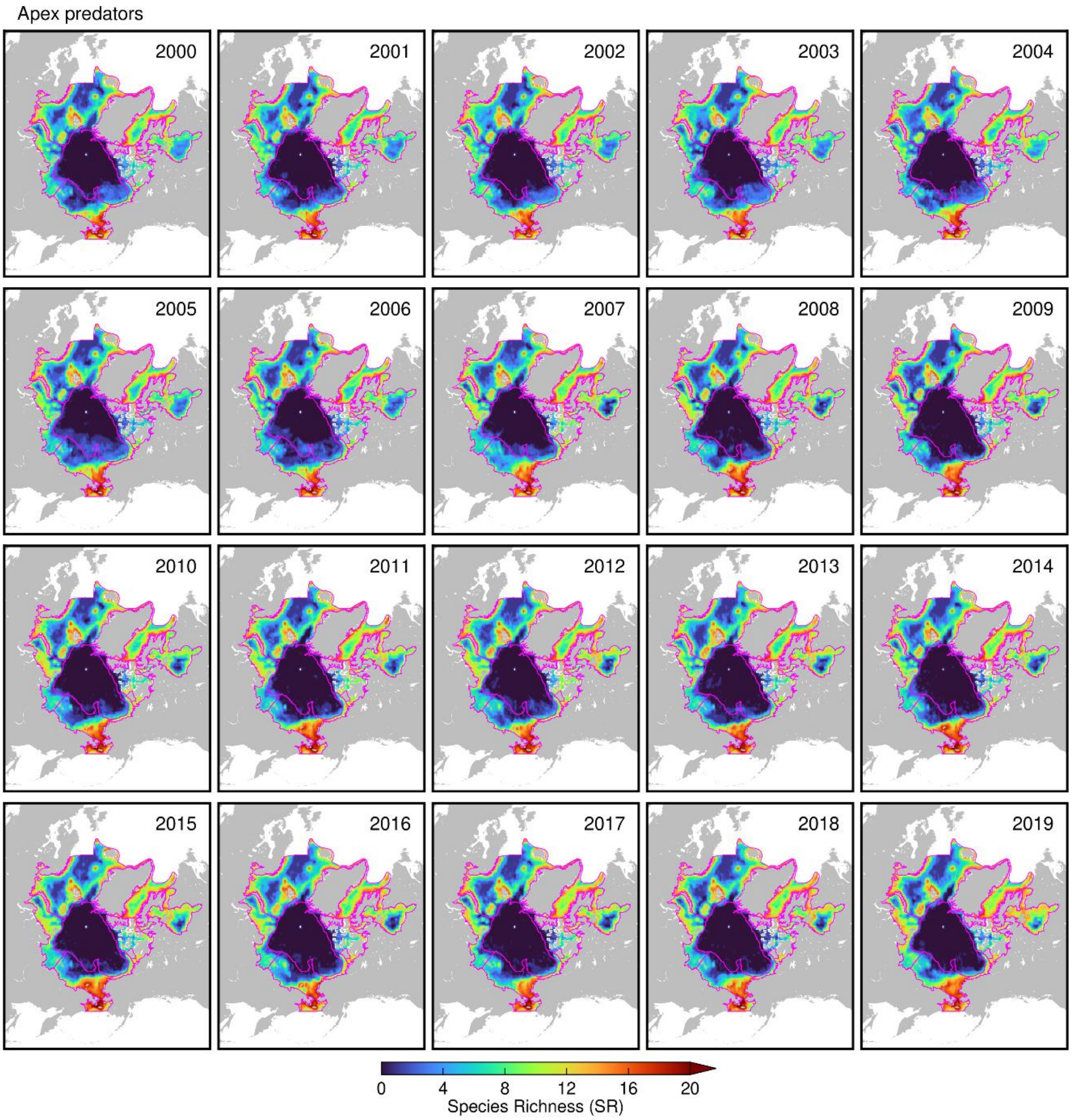

Figure S2. Spatial distributions of species richness for apex predators from 2000-2019. The maps were created using GMT 6.3.0 (https://docs.generic-mapping-tools.org/6.3/gmt.html).

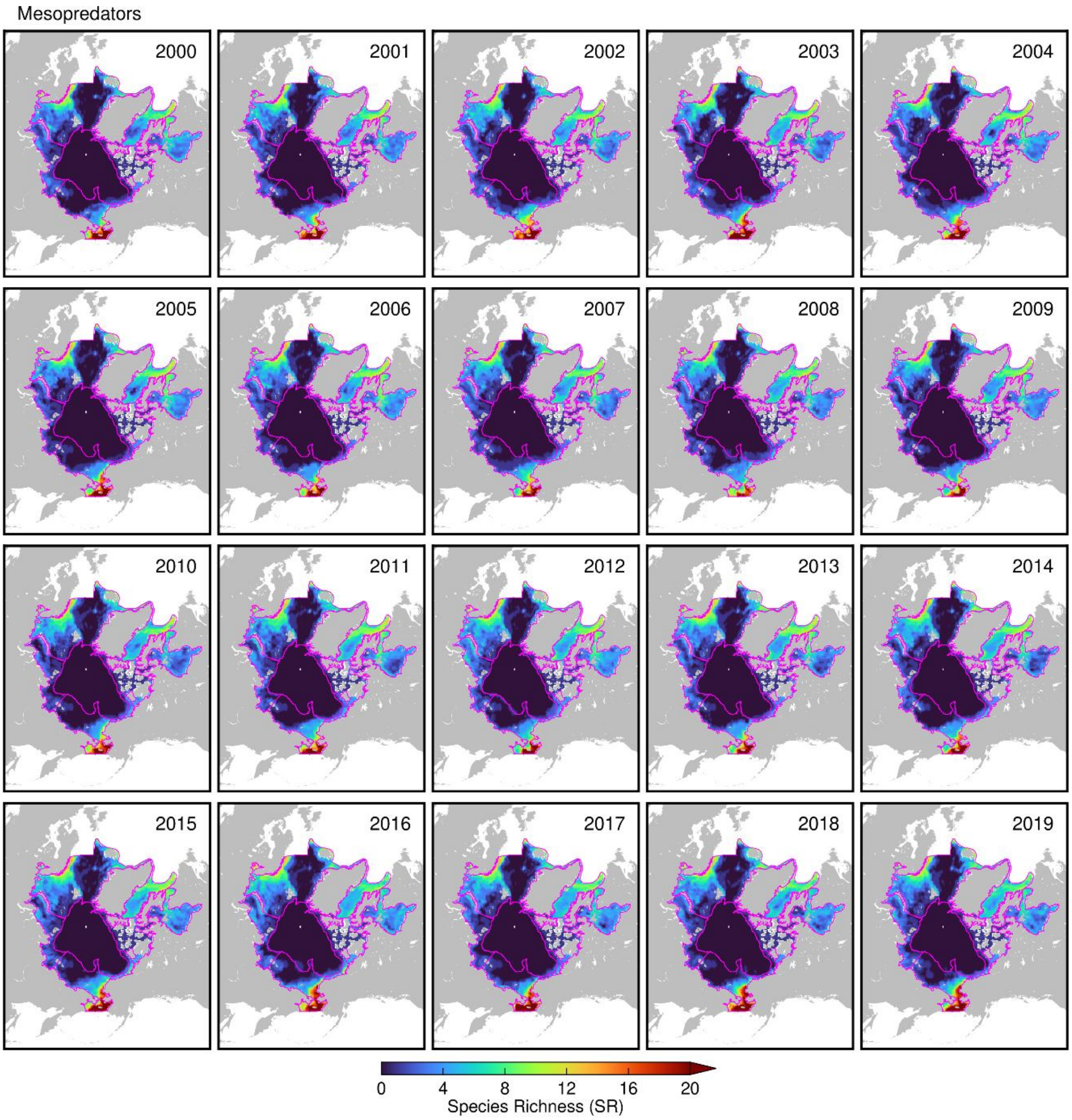

Figure S3. Spatial distributions of species richness for mesopredators from 2000-2019. . The maps were created using GMT 6.3.0 (https://docs.generic-mapping-tools.org/6.3/gmt.html).

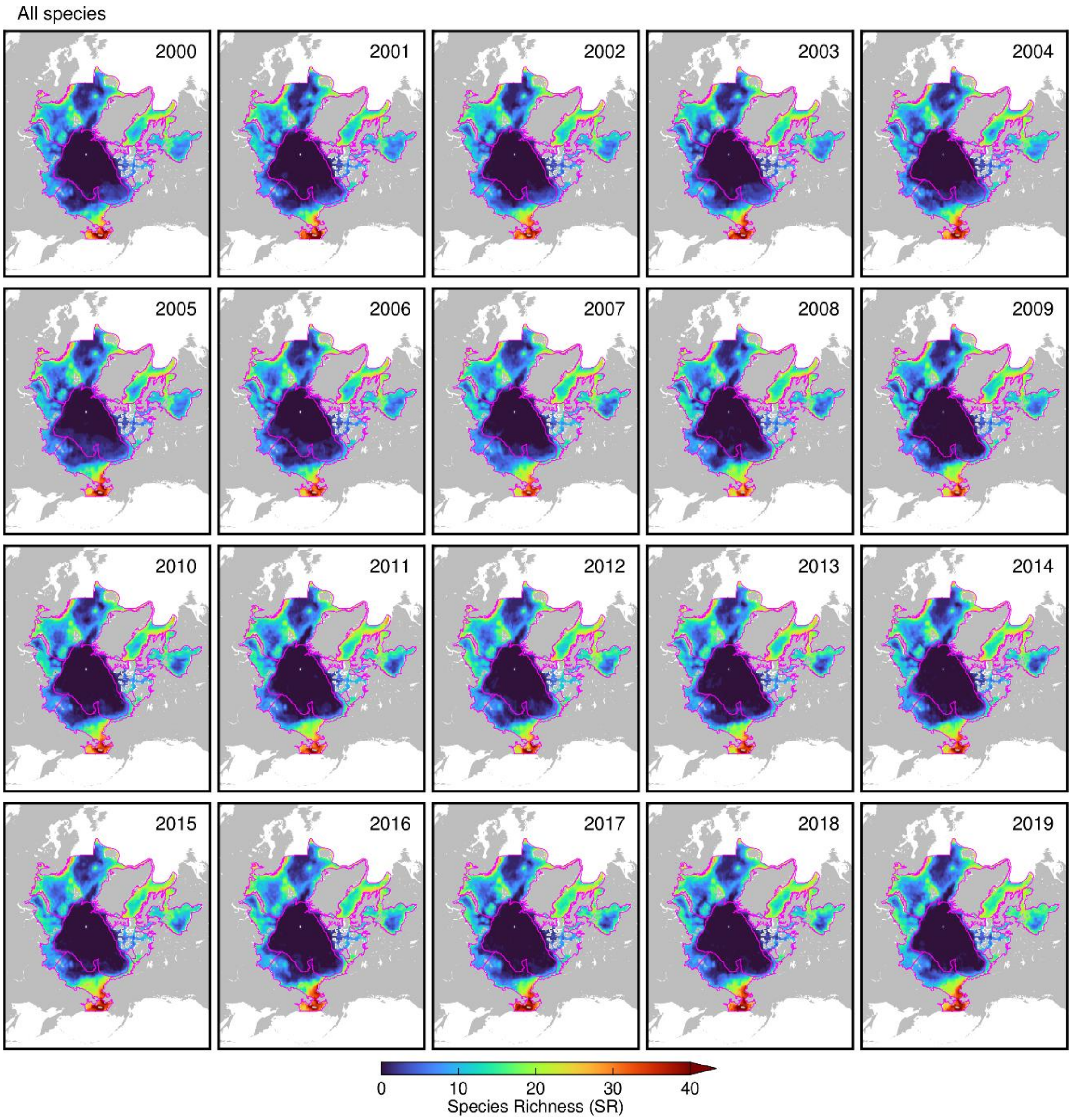

Figure S4. Spatial distributions of species richness for all species from 2000-2019. The maps were created using GMT 6.3.0 (https://docs.generic-mapping-tools.org/6.3/gmt.html).

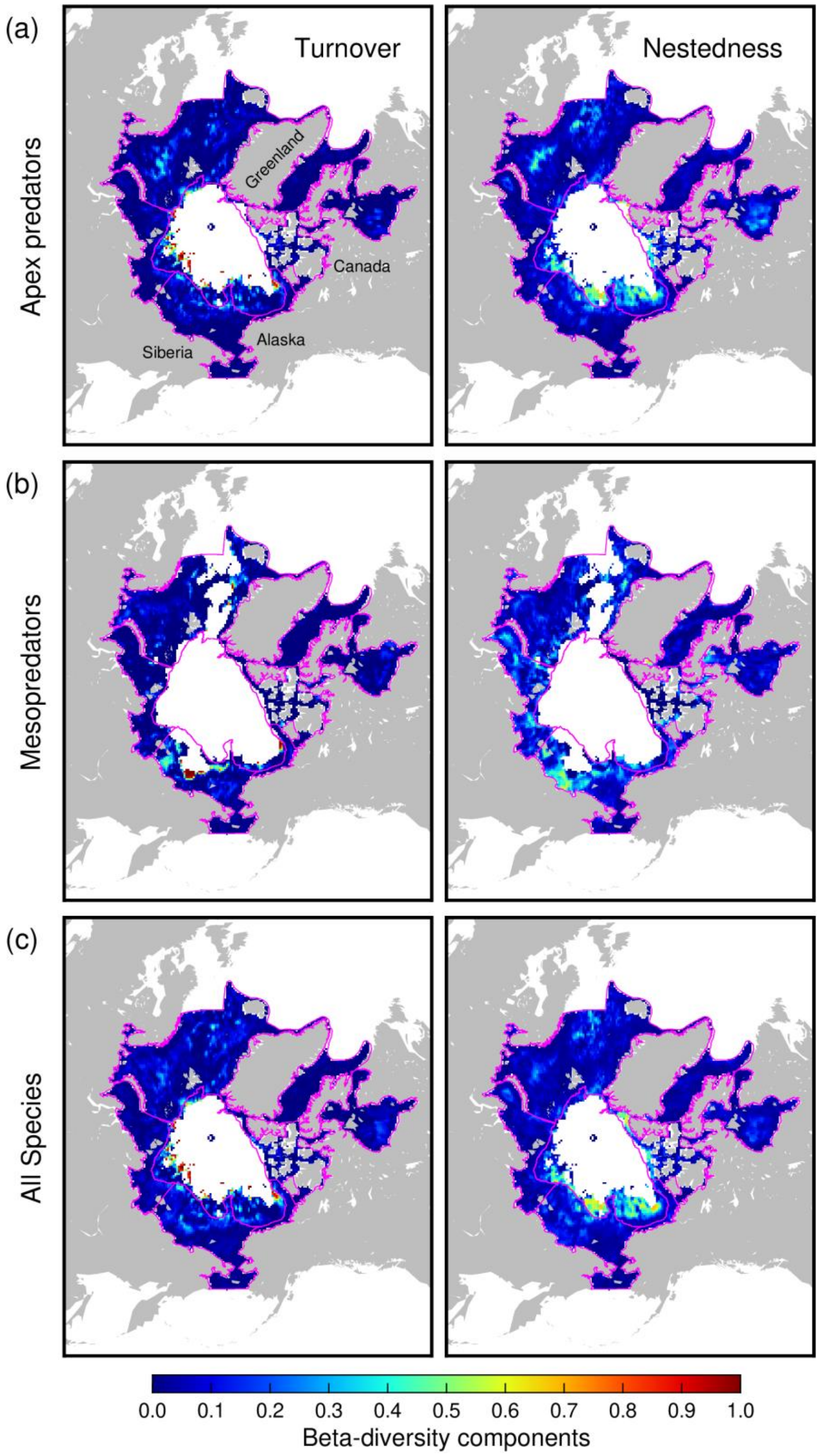

Figure S5. Turnover (left panels) and nestedness (right panels) components of beta-diversity between high and low sea ice for (a) apex predators, (b) mesopredators and (c) all species. The maps were created using GMT 6.3.0 (https://docs.generic-mapping-tools.org/6.3/gmt.html).

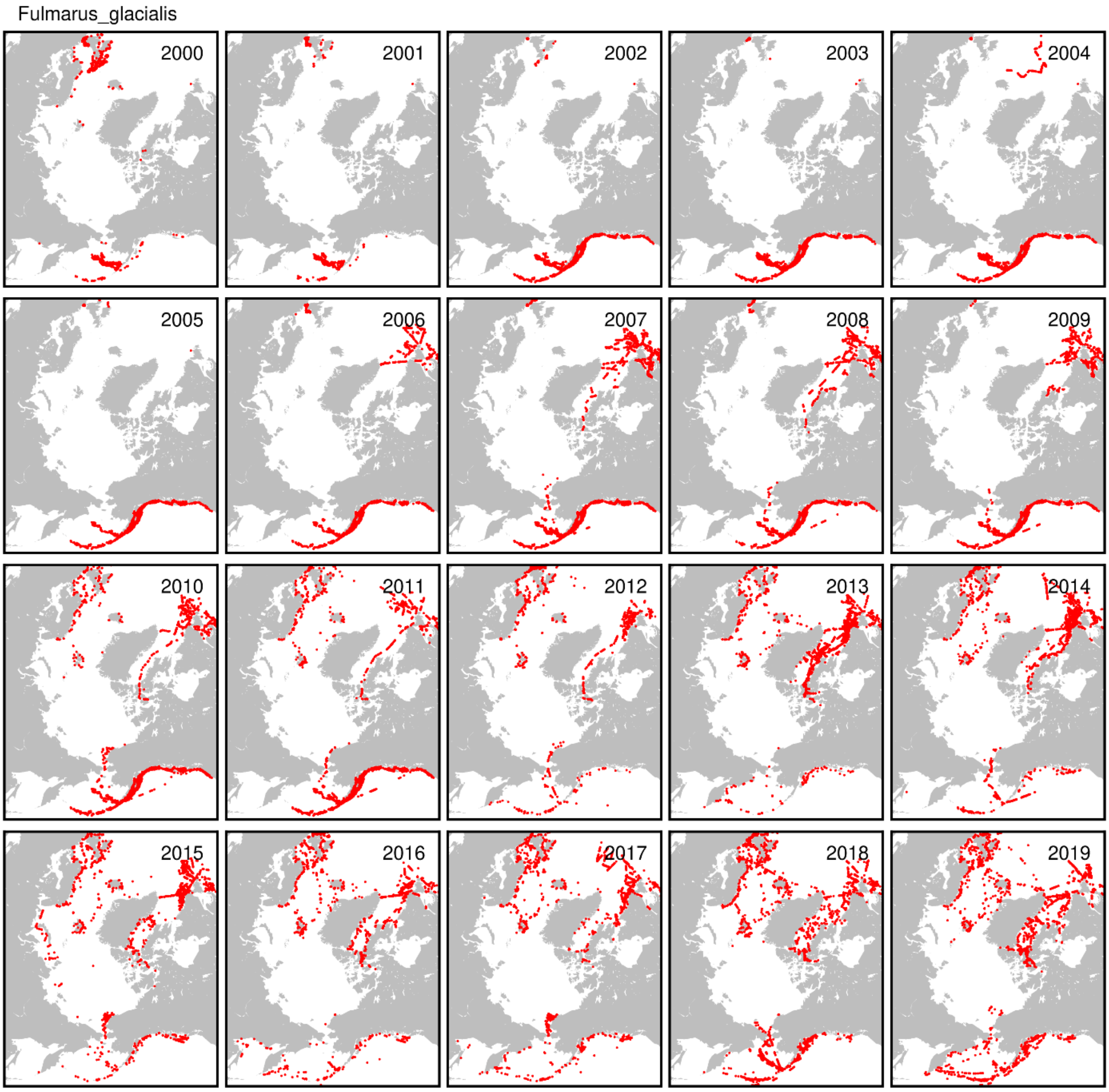

Figure S6. Spatial distributions of original and unthinned records (42,579) for species with the most number of occurrences (northern fulmar, *Fulmarus glacialis*) between 2000 and 2019. The maps were created using GMT 6.3.0 (https://docs.generic-mapping-tools.org/6.3/gmt.html).

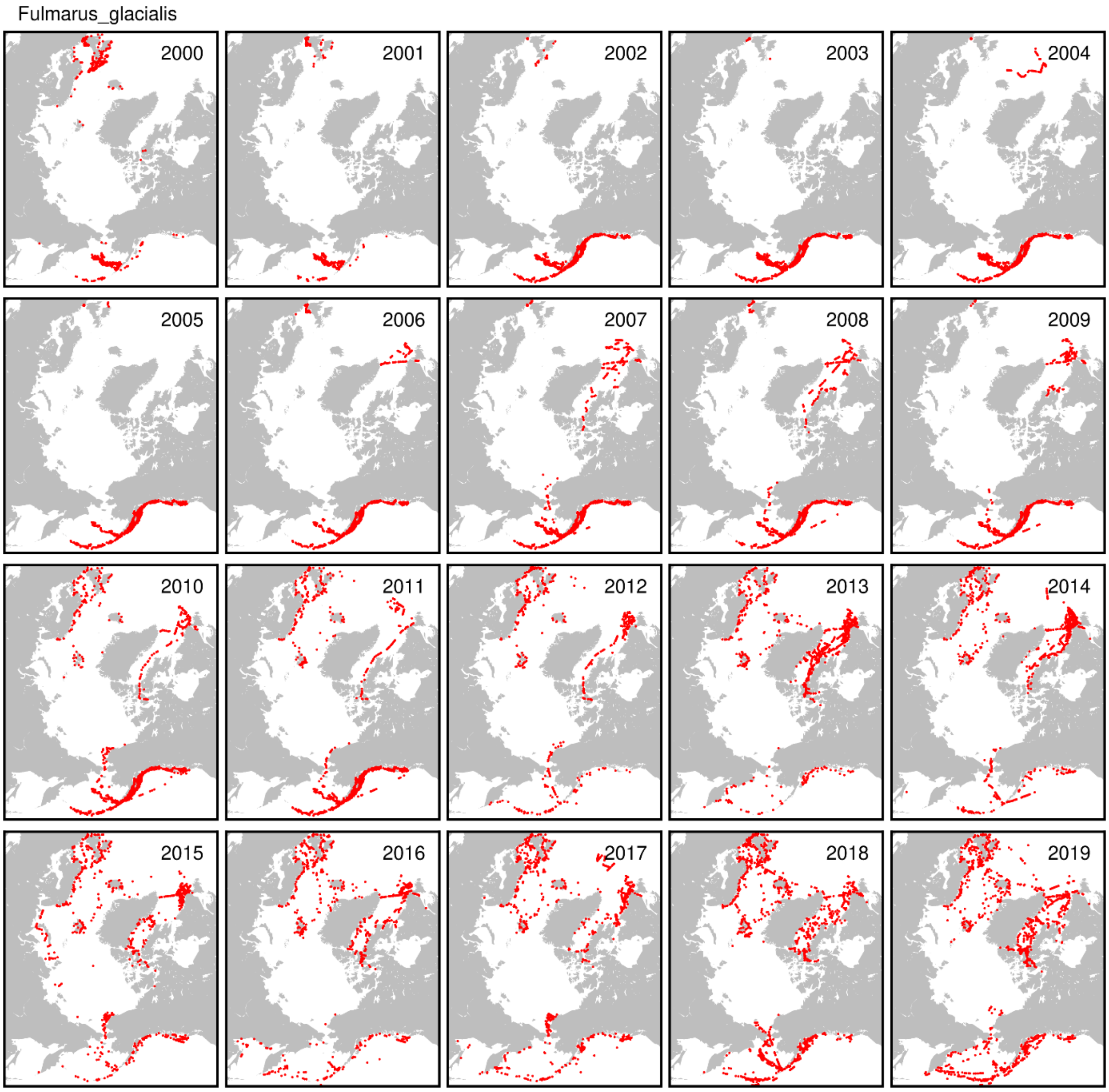

Figure S7. Spatial distributions of thinned records at 25-km cell size (11,722) for species with the most number of occurrences (northern fulmar, *Fulmarus glacialis*) between 2000 and 2019. The maps were created using GMT 6.3.0 (https://docs.generic-mapping-tools.org/6.3/gmt.html).

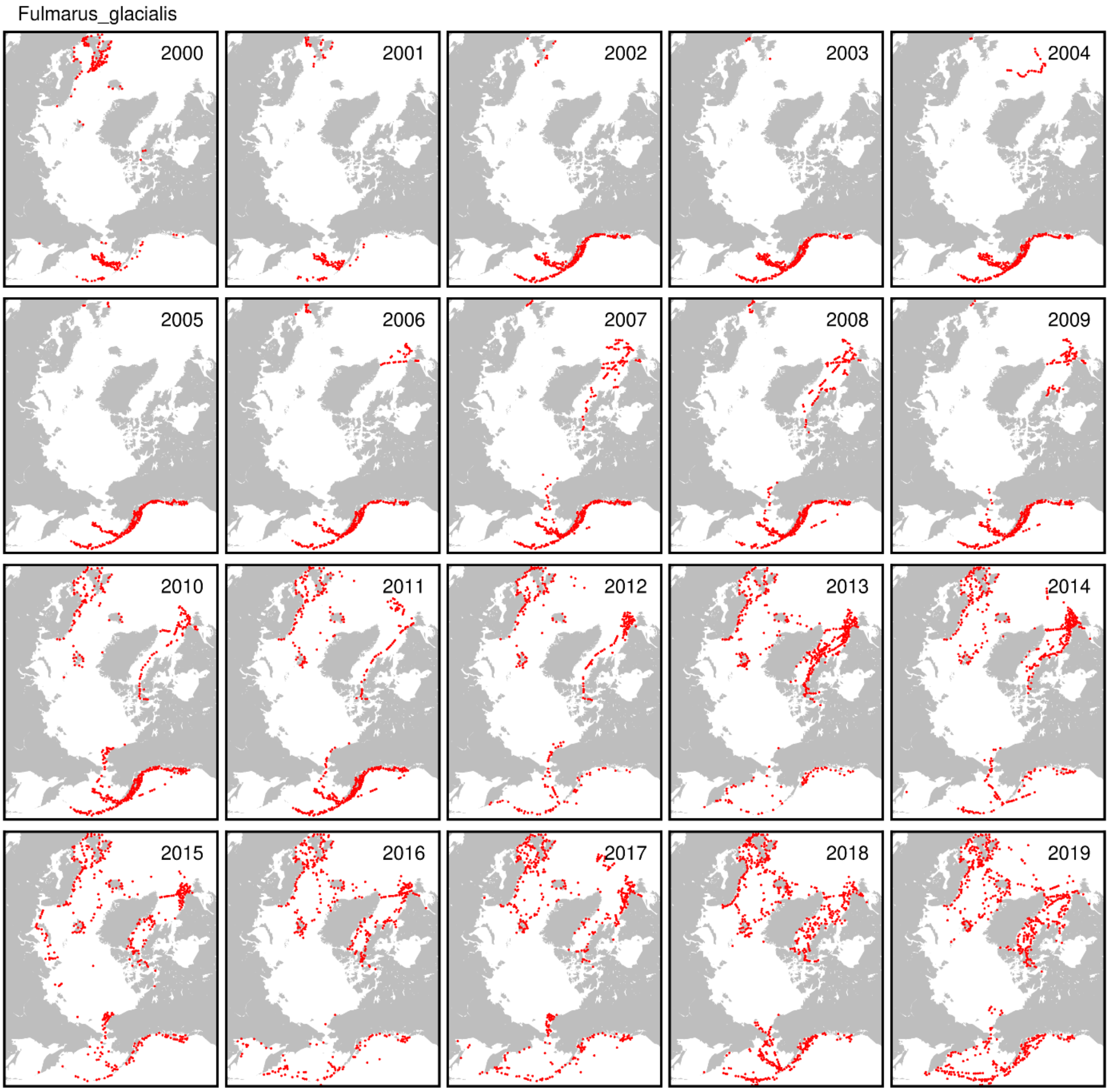

Figure S8. Spatial distributions of thinned records at 50-km cell size (6,289) for species with the most number of occurrences (northern fulmar, *Fulmarus glacialis*) between 2000 and 2019. The maps were created using GMT 6.3.0 (https://docs.generic-mapping-tools.org/6.3/gmt.html).

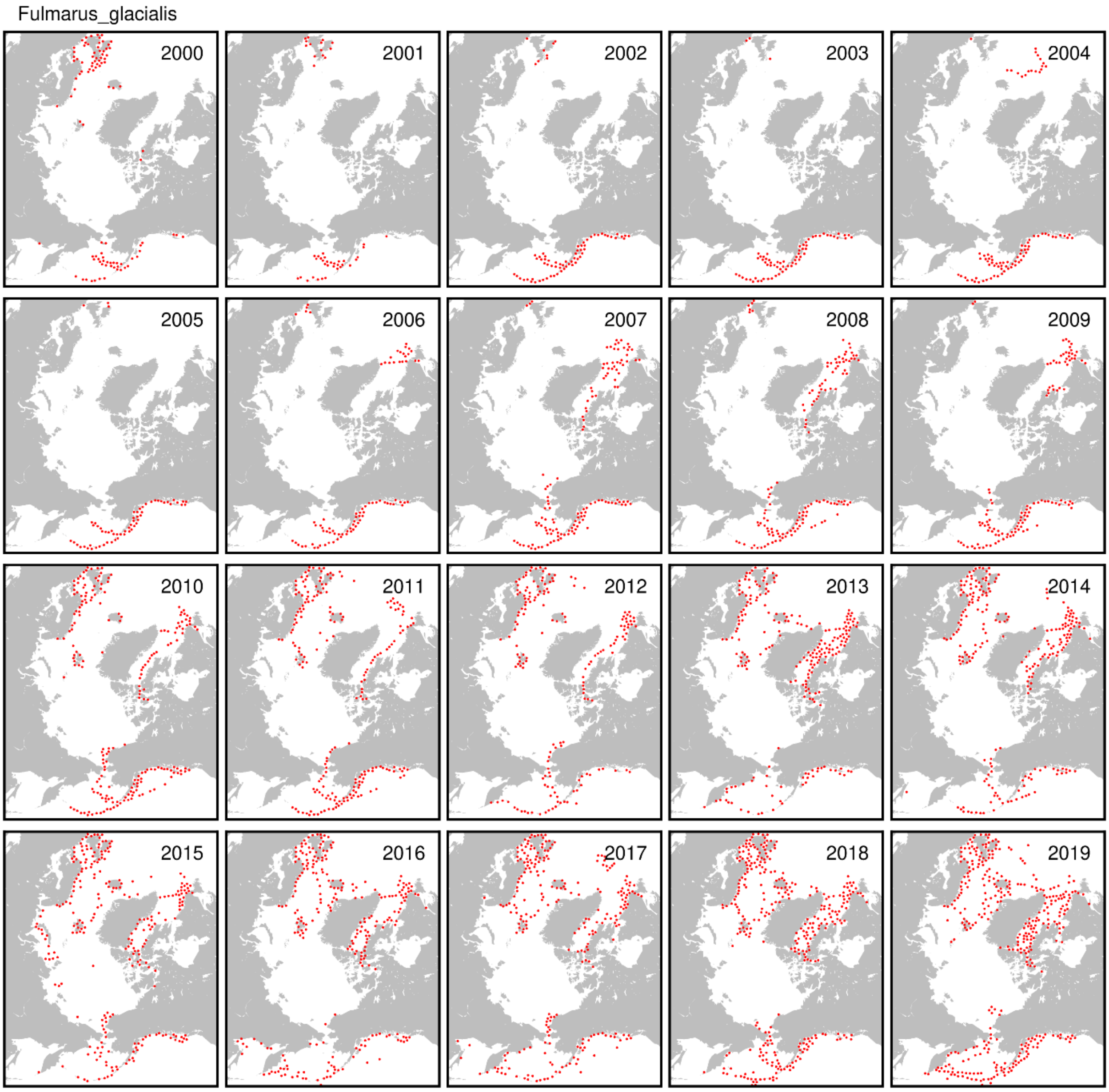

Figure S9. Spatial distributions of thinned records at 100-km cell size (3,338) for species with the most number of occurrences (northern fulmar, *Fulmarus glacialis*) between 2000 and 2019. The maps were created using GMT 6.3.0 (https://docs.generic-mapping-tools.org/6.3/gmt.html).

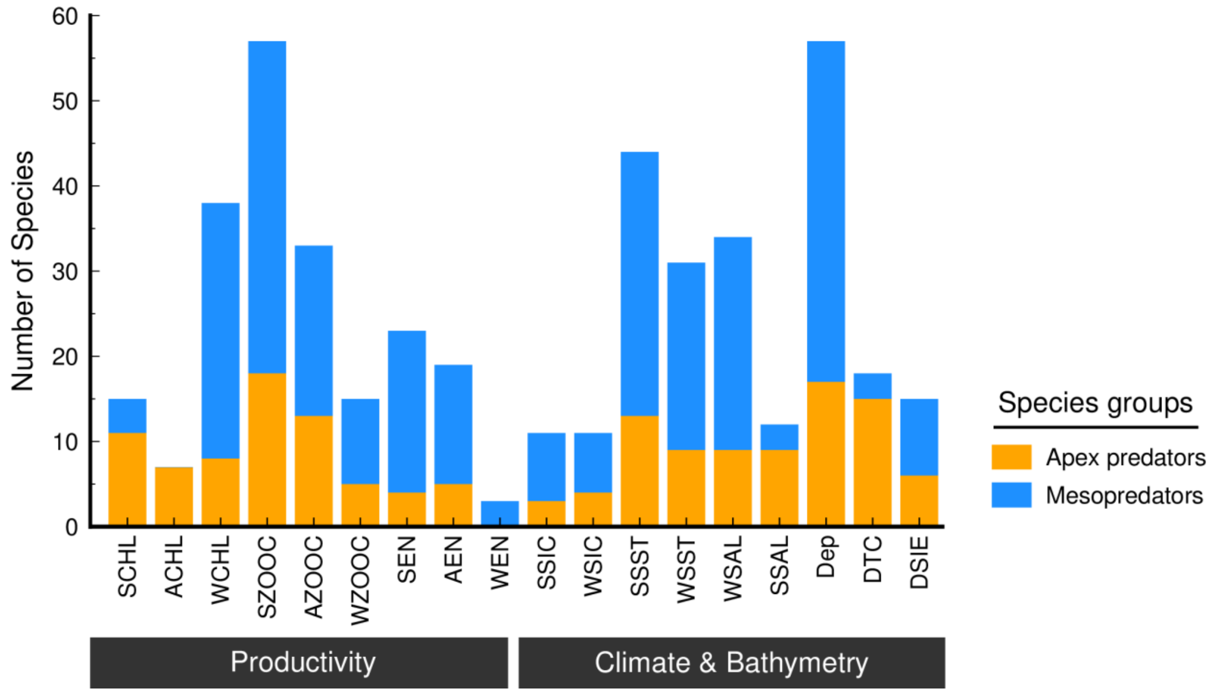

Figure S10. Number of species belonging to apex (orange bars) and mesopredators (blue bars) selecting specific environmental parameters for the final species distribution models.

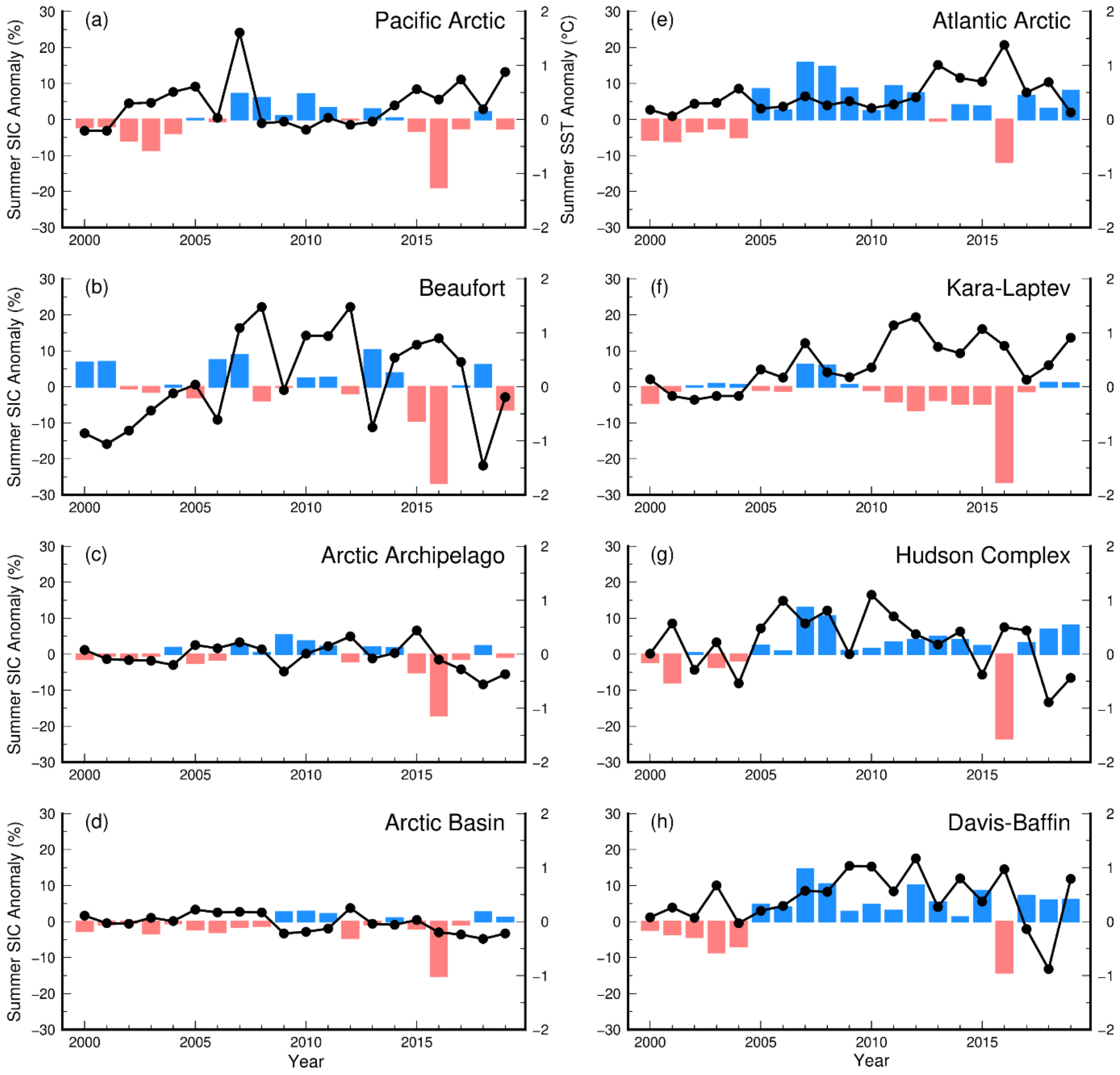

Figure S11. Region-specific annual summer sea ice concentration (colored bars) and summer sea surface temperature (SST; solid lines and circles) anomalies from 2000 to 2019 computed relative to the 30-year average for each parameter (1982-2011).
